# Genomic Surveillance of SARS-CoV-2 Lineages Indicates Early Circulation of P.1 (Gamma) Variant of Concern in Southern Brazil

**DOI:** 10.1128/spectrum.01511-21

**Published:** 2022-02-16

**Authors:** Priscila Lamb Wink, Rafaela Ramalho, Francielle Liz Monteiro, Fabiana Caroline Zempulski Volpato, Julia Biz Willig, Otávio von Ameln Lovison, Alexandre Prehn Zavascki, Afonso Luís Barth, Andreza Francisco Martins

**Affiliations:** a LABRESIS–Laboratório de Pesquisa em Resistência Bacteriana, Hospital de Clínicas de Porto Alegre, Porto Alegre, Rio Grande do Sul, Brazil; b Laboratório de Diagnóstico de SARS-CoV-2, Hospital de Clínicas de Porto Alegre, Porto Alegre, Rio Grande do Sul, Brazil; c Programa de Pós-Graduação em Ciências Farmacêuticas, Universidade Federal do Rio Grande do Sul, Porto Alegre, Rio Grande do Sul, Brazil; d Bioinformatics Core, Hospital de Clínicas de Porto Alegre, Porto Alegre, Rio Grande do Sul, Brazil; e Laboratório de Diagnóstico Molecular, Universidade Franciscana, Santa Maria, Rio Grande do Sul, Brazil; f Programa de Pós-Graduação em Ciências Médicas, Universidade Federal do Rio Grande do Sul, Porto Alegre, Rio Grande do Sul, Brazil; g Infectious Diseases Service, Hospital de Clínicas de Porto Alegre, Porto Alegre, Rio Grande do Sul, Brazil; h Internal Medicine Department, Universidade Federal do Rio Grande do Sul, Porto Alegre, Rio Grande do Sul, Brazil; i Departamento de Microbiologia, Imunologia e Parasitologia, Instituto de Ciências Básicas, Universidade Federal do Rio Grande do Sul, Rio Grande do Sul, Brazil; University of Georgia

**Keywords:** COVID-19, gamma, genomic surveillance, P.1 lineage, SARS-CoV-2

## Abstract

The SARS-CoV-2 P.1 lineage emerged in Amazonas (AM), North Brazil and its evolution has been dynamically reported associated with increased transmissibility and/or immune evasion. Here, we evaluated the lineages circulating in 29 cities in Rio Grande do Sul (RS), Southern Brazil between March 2020 and May 2021 and investigated the genetic events associated with the emergence of the P.1. A total of 202 oro/nasopharyngeal SARS-CoV-2 specimens from patients during routine hospital care were submitted to whole-genome sequencing. Phylogenetic and Bayesian Evolutionary Analyses of the P.1 lineage were carried out to determine the relationship between sequences from RS and AM and dated their common ancestor and origin. One hundred six (53%) sequences were assigned as P.1 and most carried the 22 lineage-defining mutations. All the P.1 sequences included other important mutations, such as P314L and R203K/G204R, and revealed a high genetic diversity in the phylogenetic tree. The time-scaled inference suggests that the oldest P.1 sequences from different Brazilian states share a ancestor with those from AM, but the origin of some sequences from RS is unknown. Further, the common ancestor of sequences from RS is dated to mid-June/July 2020, earlier than those previously reported from AM. Our results demonstrate that there is a high degree of genetic diversity among P.1 sequences, which suggests a continuous evolution and community spread of the virus. Although the first P.1 outbreak was reported in AM, the lineage was associated with multiple introductory events and had already been circulating in Southern Brazil prior to November 2020.

**IMPORTANCE** The SARS-CoV-2 P.1 lineage is associated with increased transmissibility and/or immune evasion and presents a dynamic evolution in Brazil. The significance of our research relies in the fact that we evaluated the SARS-CoV-2 lineages circulating in Southern Brazil between March 2020 and May 2021. This evaluation allowed us to detect the genetic events associated with the emergence of the P.1 and its sublineages. This study is important because we were able to establish that the common ancestor of P.1 sequences from Rio Grande do Sul, Southern Brazil, is dated of mid-June/July 2020, earlier than the P.1 sequences previously reported from Amazonas (AM) state. Noteworthy, the high degree of genetic diversity among P.1 sequences found in this study suggests a continuous evolution and community spread of the virus. Moreover, the oldest P.1 sequences from different Brazilian states share a ancestor with those from AM.

## INTRODUCTION

The World Health Organization (WHO) declared COVID-19 a public health emergency of international interest in 2020 (1). In fact, since the emergence of the severe acute respiratory syndrome coronavirus 2 (SARS-CoV-2), many different lineages of SARS-CoV-2 have been reported worldwide (2). The causative agent of COVID-19 proved to present high genetic diversity as it adapted and evolved quickly (2).

In Brazil, a Variant of Concern (VOC) P.1, also known as Gamma (3), emerged at the beginning of December 2020 and was associated with the second wave of infections in Manaus, the capital of the state of Amazonas (AM), North Brazil (4, 5). The P.1 lineage was first detected in four travelers returning to Japan from AM at the beginning of January 2021 (6). As soon as it was recognized as an emerging lineage, P.1 was confirmed in six Brazilian states in late February 2021 (4). According to Faria et al. (4), the common ancestor of the P.1 lineage emerged around November 15, 2020, in Manaus, 3–4 weeks before the first confirmed cases of this lineage in the region. Similarly, Naveca et al. (5), using Bayesian reconstruction and phylogeographic analyses, estimated that VOC P.1 arose in Manaus around late November 2020 and rapidly spread to other municipalities and neighboring countries.

In the state of Rio Grande do Sul (RS), Southern Brazil, the first report of the P.1 lineage was of a clinical specimen obtained on February 1st, 2021, from a patient in the city of Gramado who had no history of traveling or contact with any returning travelers (7). Subsequent to this first report, Silva et al. (8) described an infection caused by P.1 on November 30, 2020, in RS, 3 months earlier than the first evidence of this lineage in the state. However, the authors inferred that P.1 probably only started to circulate in RS later because they were unable to identify any other P.1 sequences between November 2020 and January 2021 in the region (8). Noteworthy, there was a high incidence of P.1 lineage in RS in February 2021, which coincided with a significant increase in hospitalizations (9).

A phylogenetic analysis indicated that the VOC P.1 descended from the B.1.1.28 lineage (4), which together with the B.1.1.33 lineage were the first dominant lineages in Brazil in 2020 (5). These lineages were gradually replaced by the P.2 Variant of Interest (VOI), which was later replaced by the P.1 lineage (4). The replacement by P.1 was an abrupt process in AM, since it became the dominant lineage in the region in less than 2 months (5).

The P.1 lineage was defined by 17 amino acid changes (including 10 in the Spike protein), three deletions, four synonymous mutations, and one four-base-pair nucleotide insertion compared to its immediate ancestor B.1.1.28 (4). The P.1 lineage-defining mutations in the Spike protein, especially in the receptor-binding domains (RBD) such as K417T, E484K and N501Y, are of concern because they may enhance ACE2 affinity and contribute to antibody evasion (10). Recently, new sublineages (P.1.2 to P.1.8) that have emerged from P.1 have been reported in Brazil (11, 12), which indicates a continuous evolution of the virus and the community spread in the country.

The aim of this study was to evaluate the SARS-CoV-2 lineages circulating in RS state, southernmost Brazil, from March 2020 to May 2021. We also investigated the genetic events associated with the emergence of the VOC P.1 and its sublineages in the region.

## RESULTS

### Epidemiological data.

From the 202 SARS-CoV-2 specimens sequenced, 200 high quality sequences (<6% Ns, >29.8 Kb) were included in the analysis. The specimens obtained during the year 2020 (*n* = 74; Ct value range: 9.39–25.94) belonged to patients aged between 2 months and 82 years old (median age = 52 years). The specimens from 2021 (*n* = 126; Ct value range: 9.0–25.99) originated from patients aged between 2 months and 65 years old (median age = 43 years) (Table S1). Most of the specimens (*n* = 134, 67%) were obtained from patients living in Porto Alegre city, the capital of RS; 50 (25%) specimens were from other cities in the metropolitan region of Porto Alegre; and 16 (8%) were from 17 other cities in RS (Table S1; Fig. S1-A) (13).

### The distribution of SARS-CoV-2 lineages and mutations found in VOC P.1.

The lineages identified in the year 2020 and from January to May 2021 are represented in Fig. S1-B. Of the 74 specimens from 2020, most of them were classified into five SARS-CoV-2 lineages: B.1.1.28 (*n* = 25; 34%), B.1.1.161 (*n* = 25; 34%), B.1.1 (*n* = 6; 26%), B.1.1.33 (*n* = 4; 5%), and B.1.91 (*n* = 4; 5%) (Table S1). It is important to highlight that we identified only one sequence assigned as P.1 from November 2020. Of the 126 specimens sequenced from 2021, most of them were classified as P.1 (*n* = 97; 77%), followed by P.2 (*n* = 12; 9.5%), P.1.2 (*n* = 6; 4.7%), B.1.1.28 (*n* = 6; 4.7%), and P.1.1 (*n* = 2, 1.5%) (Table S1; Fig. S1).

Of the 106 P.1 sequences (98 P.1, six P.1.2, two P.1.1), 99 (93.4%) presented most of the 22 P.1 lineage-defining mutations, with exception of one synonymous mutation in the ORF1ab (C12778T) and one missense mutation in the Spike (R190S), which were present in 44 (41.5%) and 79 (74.5%) sequences, respectively (Table 1). In addition, five P.1 nondefining mutations were found in all of the P.1 sequences: two in the ORF1ab (C3037T and P314L) and three in the Nucleocapsid (A28877T, G28878C and R203K/G204R). Another P.1 nondefining mutation was found in the Spike gene (D614G, *n* = 41, 38.7%). Of these six P.1 nondefining mutations, three were classified as missense and resulted in amino acid substitutions (Table 1). Noteworthy, two sequences did not present the P.1 lineage-defining mutation N501Y in the Spike protein, RBD domain.

**TABLE 1:** Detailed description and frequency of mutations found in the P.1 lineage and sublineage sequences^*a*^

Genomic position	Effect	Gene/region	Amino acid change	Frequency (%)
**C241T**	Intergenic	5’UTR	-^*b*^	106 (100.0)
**T733C**	Synonymous	ORF1ab	-	104 (98.1)
**C2749T**	Synonymous		-	105 (99.0)
C3037T	Synonymous		-	106 (100.0)
**C3828T**	Missense		S1188L	104 (98.1)
A5648C	Missense	*nsp3*	K1795Q	106 (100.0)
del11288-11296	NA		-	106 (100.0)
C12778T	Synonymous	*nsp9*	-	44 (41.5)
C13860T	Synonymous		-	105 (99.0)
C14408T	Missense	*RdRp*	P314L	106 (100.0)
G17259T	Missense	*Helicase*	E1264D	105 (99.0)
C21614T	Missense	Spike	L18F	99 (93.4)
C21621A	Missense		T20N	105 (99.0)
C21638T	Missense		P26S	104 (98.1)
G21974T	Missense		D138Y	106 (100.0)
G22132T	Missense		R190S	79 (74.5)
A22812C	Missense		K417T	106 (100.0)
G23012A	Missense		E484K	106 (100.0)
A23063T	Missense		N501Y	104 (98.1)
A23403G	Missense		D614G	41 (38.7)
C23525T	Missense		H655Y	105 (99.0)
C24642T	Missense		T1027I	103 (97.2)
G28167A	Missense	ORF8	E92K	105 (99.0)
ins28263-28266	NA		-	106 (100.0)
C28512G	Missense	Nucleocapsid	P80R	106 (100.0)
A28877T	Synonymous		-	106 (100.0)
G28878C	Synonymous		-	106 (100.0)
GGG2888128883AAC	Missense		R203K/G204R	106 (100.0)

a Mutations found in P.1 (*n* = 98), P.1.1 (*n* = 2) and P.1.2 (*n* = 6) sequences. P.1 lineage-defining mutations are highlighted in bold. Only mutations observed in more than 40 genomes from this study are shown. del: nucleotide deletion; ins: nucleotide insertion; NA: not applicable; ORF: open reading frame; nsp: nonstructural protein; RdRp: RNA-dependent RNA polymerase; UTR: untranslated region. The proteins nsp3, nsp9, RdRp, and helicase belong to the ORF1ab. Mutations shared with the B.1.1.28 lineage are not described. All the amino acid mutations are based on Nextclade (https://clades.nextstrain.org).

b -, non-amino acid change.

From the two specimens assigned as a P.1.1 sublineage by Pangolin, the specimen 76_LABRESIS presented all the 22 P.1 lineage-defining mutations while the 77_LABRESIS specimen lacked the T733C, C21614T (L18F), and G22132T (R190S) mutations. Five specimens (6_LABRESIS, 101_LABRESIS, 195_LABRESIS, 198_LABRESIS, and 211_LABRESIS) were assigned as a P.1.2 sublineage, as they had two synonymous defining mutations (C1912T, and C28789T) tree missense defining mutations (D762G, T1820I, D155Y). Other specimen assigned as P.1.2 (90_LABRESIS) had all of them except the T1820I mutation. From the five P.1.2 sublineage-defining mutations, three resulted in amino acid substitutions: one in the ORF1ab (D762G/A2550G), one in the ORF3a (T1820I/C5724T), and one in the Nucleocapsid gene (D155Y/G25855T). Our data demonstrated that the first occurrence of P.1.1 and P.1.2 was in February 2021, 3 months after the parental P.1 was detected in the region (Fig. S1 and Table S1).

### Phylogenetic analyses.

**FIG 1:**
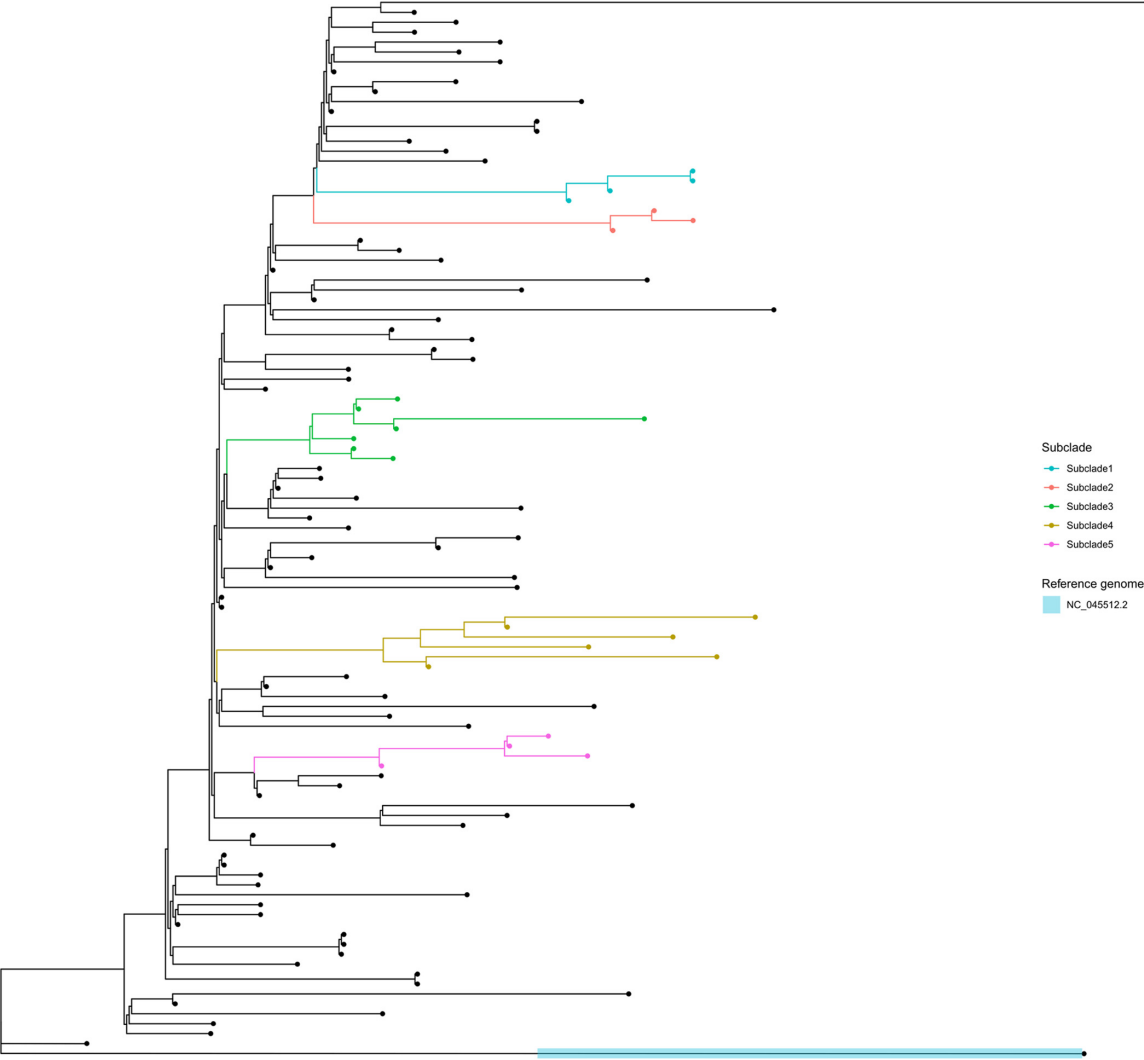
Maximum likelihood phylogenetic tree (ML, GTR, 1,000 bootstrap) of the 106 P.1 sequences obtained in this study. The scale of the phylogenetic branches is based on nucleotide substitution per site. Subclade 1 (blue) grouped together four sequences (171_LABRESIS, 177_LABRESIS, 179_LABRESIS, and 184_LABRESIS), Subclade 2 (red) grouped together three sequences (187_LABRESIS, 197_LABRESIS, and 201_LABRESIS), Subclade 3 (green) grouped together seven sequences (5_LABRESIS, 13_LABRESIS, 73_LABRESIS, 98_LABRESIS, 104_LABRESIS, 105_LABRESIS, and 212_LABRESIS), Subclade 4 (yellow) grouped together six sequences belong to P.1.2 sub-lineage (6_LABRESIS, 90_LABRESIS, 101_LABRESIS, 195_LABRESIS, 198_LABRESIS, and 211_LABRESIS) and Subclade 5 (pink) grouped together four sequences (11_LABRESIS, 31_LABRESIS, 34_LABRESIS and 41_LABRESIS). Reference genome (blue highlight): NC_045512.2.

The phylogenetic tree of the P.1 lineage demonstrated a high degree of genetic diversity among these 106 sequences (Fig. 1). Forty-seven sequences belonged to a single, distinct monophyletic clade, collected between January and May 2021. In addition, it was possible to observe exclusive mutations for five defined small subclades. The subclade 1, comprising sequences that carried the mutations C2973T (A903V), A4314G (K1350R), C5796T (T1844I), C12439T, and G18816T in the ORF1ab and C25516T (P42S) in the Spike protein. The subclade 2, comprising sequences that carried the mutations T8908C, C11882T (L3873F) in the ORF1ab, A22991T, G22992C, G25305T (C1248F) in the Spike, and G28287T (G5V) in the Nucleocapsid. The subclade 3, comprising sequences that carried the mutations C1420T and G5063A (D1600N) in the ORF1ab. The subclade 4 comprised the P.1.2 sequences mentioned above. The subclade 5, comprising sequences that carried the mutations C1545T (A427V) in the ORF1ab, C23413T and G24872T (V1104L) in the Spike. Three sequences from the subclade 5 presented one more mutation in the ORF1ab (G10979A-V3572M). The P.1.1 sequences were not grouped as a subclade.

### Bayesian evolutionary analysis of P.1 lineage.

To better understand the emergence and evolution of the P.1 lineage in RS, we used molecular clock phylogenetic analysis. The exploratory analysis of root-to-tip distances for the P.1 (data set A, C and D) and B.1.1.28 (data set B) lineages revealed an evolutionary rate of 1.5865 × 10^−3^ substitutions/site/year. The estimated date of the root time to the most recent common ancestor (tMRCA) was around 25 May, 2020 with a correlation coefficient of 0.7867 (Fig. S2).

The molecular clock (Fig. S3) demonstrated that P.1 sequences were grouped into a single well-supported clade (posterior probability = 0.99) and a common ancestor dated to around 28 May, 2020 (95% HPD: 24 January to 26 June 2020). The common ancestor date of the specimen 164_LABRESIS (EPI_ISL_3233232), collected on 29 November 2020 in RS (sequenced in this study), was estimated to be around 20 June, 2020 (95% HPD: 28 Abril to 26 August 2020), and the specimen RS-LMM38991 collected on 30 November 2020 in RS (EPI_ISL_2241496) to around 25 July, 2020 (95% HPD: 8 June to 4 August 2020).

**FIG 2:**
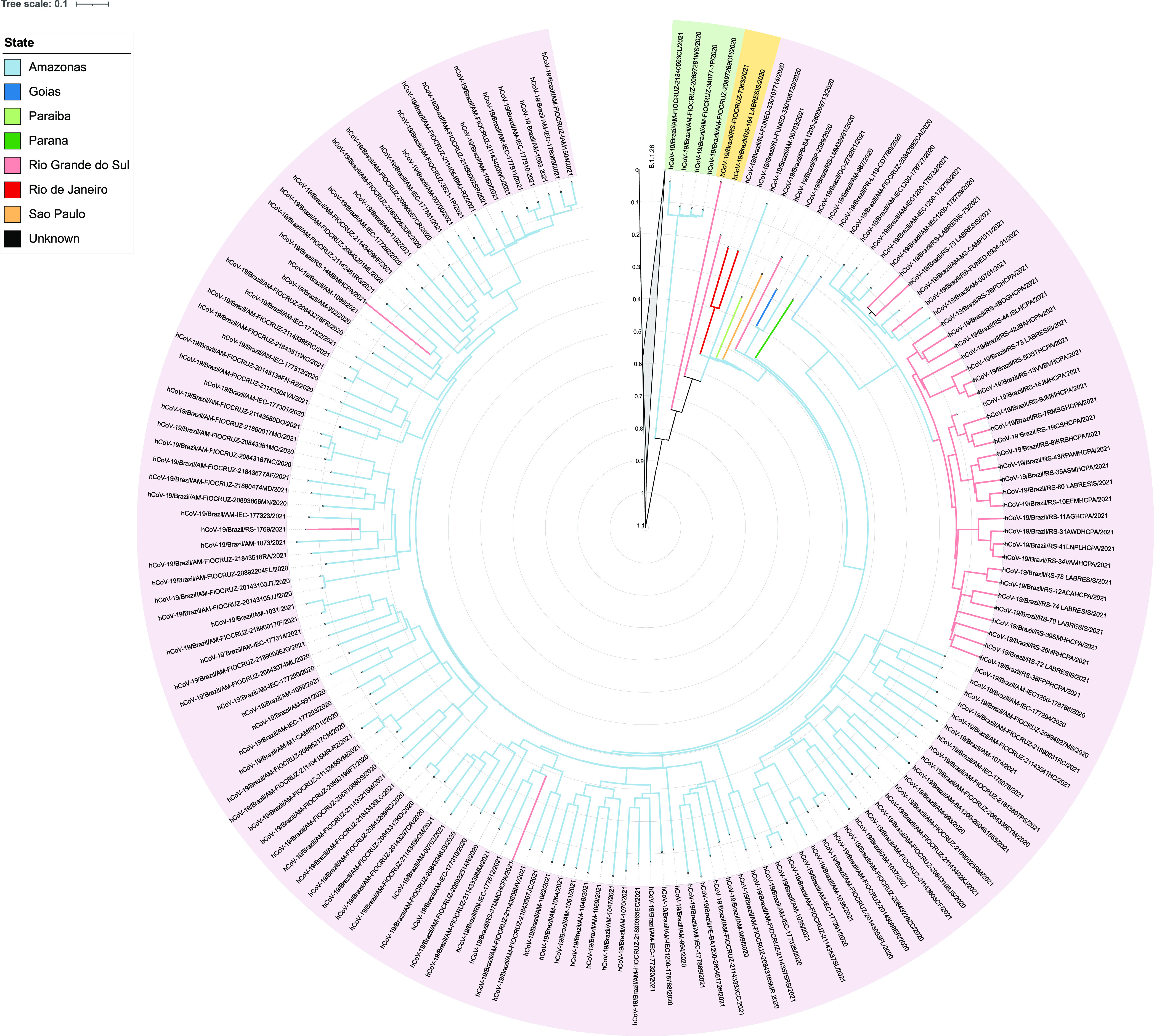
Time-scaled and ancestral character reconstruction of B.1.1.28 (data set B) and P.1 lineages (data set A, C, and D). The clade of the B.1.1.28 lineage was collapsed for better visualization. The outer circle is color-coded according to the lineage. Branches are colored according to the sampling location and the most probable location state of their descendant nodes, as indicated in the legend. The sequences highlighted in purple and green colors share a ancestor node of Amazonian origin. The sequences highlighted in yellow color share a ancestor node of unknown origin. The axis and grid represent the divergence time of the sequences in years.

The PastML analysis demonstrated that the P.1 sequences (data sets A, C and D) belonged to the major clade with well-defined three genomic diversity events (Fig. 2). The sequences highlighted in purple (*n* = 162) and green (*n* = 4), from different states of Brazil, share a original ancestor from Amazonas. However, the sequences highlighted in yellow from the state of RS (*n* = 2) presented different ancestor nodes of unknown origin.

## DISCUSSION

In this study, we analyzed 200 SARS-CoV-2 genomes from RS, Southern Brazil, from 2020 and 2021, mainly from Porto Alegre city (capital of RS) and its metropolitan region. Our data demonstrate that the lineages of SARS-CoV-2 circulating in 2020 were gradually replaced by the VOC P.1 which has become the most prevalent lineage in RS since February 2021 (9), as well as in some other Brazilian regions (4, 5, 12).

According to our results, most of the P.1 sequences carried the 22 lineage-defining mutations, including the three mutations in the Spike protein, RBD domain (K417T, E484K and N501Y) associated with evasion of the immune system and greater transmissibility (10). Moreover, all the P.1 sequences accumulated other important mutations, such as P314L in the ORF1ab and R203K/G204R in the Nucleocapsid gene. Furthermore, 41 (38.7%) sequences presented the D614G mutation in the Spike gene and, after its first description in February 2020, it has been identified in all VOCs (14). These mutations, in addition to the 5′UTR:241 mutation, appear to dominate the most frequent nonlineage-defining mutations worldwide and their stability suggests virus adaptation to humans (15).The phylogenetic tree of the P.1 sequences obtained from this study revealed a high degree of genetic diversity (Fig. 1). The sequences were grouped in several distinct clades, suggesting multiple introductions of this lineage in Southern Brazil. It was possible to identify specific mutations for five defined subclades, most of them rarely found in deposited genomes (less than 15 occasions; accessed on https://nextstrain.org/ 20 July 2021) and whose significance is unknown so far. However, the comparative phylogenetic tree indicated that six sequences (6_LABRESIS, 90_LABRESIS, 101_LABRESIS, 195_LABRESIS, 198_LABRESIS, and 211_LABRESIS) were clustered together in subclade 4, assigned as P.1.2 (Fig. 1). The P.1.2 sublineage was identified in Southern Brazil in February 2021 and later it was also detected in other Brazilian regions and other countries (11). This sublineage evolved from parental P.1 and, according to our data, its occurrence increased over time (Fig. S1; Table S1). The P.1.2 sublineage carries some different mutations in comparison to the P.1 lineage and it was considered a Variant of Interest (VOI) due to the mutation D155Y in the ORF3a, which results in a low viral affinity to the host caveolin-1 protein, thereby avoiding cell apoptosis and extending the asymptomatic phase of infection (11, 16). As reported recently, the pattern of accumulated mutations in some P.1 sublineages seems to increase the transmissibility of the virus. However, it does not necessarily increase its capability to evade immune system among vaccinated population. Consequently, these sublineages could replace parental P.1 mainly in the community with low vaccination coverage (12).

The VOC P.1 was first detected in December 2020 in Manaus city (Amazonas state), and in January 2021 the cases due to the P.1 variant represented 85.4% (41/48) of the identified SARS-CoV-2 sequences in the city (4). As soon as it was recognized as an emergent lineage in late February 2021, P.1 had been confirmed in six Brazilian states (4). The prevalence of the P.1 variant increased extremely rapid by January 2021 in Brazil and other countries (6, 12, 17). In February 2021, it reached nearly 40% of sequenced cases in South America. In the United States, the VOC P.1 first appeared in mid-January. During the last 2 weeks in April 2021, the P.1 variant represented an estimated 5.0% of U.S. SARS-CoV-2 infections, demonstrating an emergence of the P.1 VOC in the country (18). According to WHO, the P.1 (Gamma) variant has already reached 91 different countries (14).

In the RS state, the first report of the VOC P.1 was in the late January 2021 (6). After this first description in RS, Silva et al. reported that the P.1 lineage had already been circulating in the state in late November 2020 but was not disseminated among individuals in the region (7). Moreover, Naveca et al. (5) reported that the P.1 and P.1-like variants share a ancestor dated from August 2020 and suggested that P.1 was circulating in Amazonas before the outbreak (late December 2020), even though this variant was not found in specimens collected between October and November 2020.

Our findings demonstrated that the divergence time of the oldest P.1 sequence identified in Brazil (Fig. S3; dated from around 10 July, 2020) is greater than the oldest specimen from AM (AM-FIOCRUZ-20842882CA; Fig. S3; dated from around 24 November, 2020). The same result was observed for the two oldest P.1 sequences from RS, both collected in November 2020 (164_LABRESIS and RS-LMM38991), which were obtained from patients with no history of travel or contact with returning travelers from Manaus (8, 19). In fact, according to the molecular clock, the common ancestor of these sequences from RS is dated to mid-June/July, earlier than those previously reported from AM (4, 5).

Besides, the time-scaled inference (Fig. 2) provided evidence of at least 3 major genetic diversity events, related to the evolution of P.1, and suggests that the sequences with the longest divergence time from different states of Brazil share a ancestor with those from AM. However, we observed that the 164_LABRESIS and RS-FIOCRUZ-7363 sequences from RS share an ancestral node of unknown origin, which could be from AM or even from RS. These findings corroborate the idea that the P.1 lineage was introduced on multiple genetic events in the country (5) and could have arisen in other Brazilian states before Amazonas.

In conclusion, our results demonstrated a high degree of genetic diversity among the P.1 genome sequences, which suggests the continuous evolution and community spread of the virus. Although the first VOC P.1 outbreak was reported in Manaus city, the lineage had already been circulating in Southern Brazil before November 2020. Nevertheless, it is well known that the P.1 spread faster in Manaus although the reasons for this are unclear. Thereby, the monitoring of virus evolution is an important tool to understand the emergence and dissemination of the old and new SARS-CoV-2 lineages, in particular the VOCs.

## MATERIALS AND METHODS

### Sampling and next-generation sequencing.

A total of 202 SARS-CoV-2 specimens (75 from 2020 and 127 from 2021; Table S1) were submitted to whole genome sequencing (WGS). Samples were obtained from oro/nasopharyngeal swabs of patients attending a tertiary-care hospital that is a COVID-19 referral center in southern Brazil and showed a cycle threshold ≤ 25.99 in the RT-qPCR. Consensus sequences were generated by the QIASeq SARS-CoV-2 pipeline (Qiagen CLC Genomics Workbench 21) and the specimens were classified using the Phylogenetic Assignment of Named Global Outbreak Lineages (Pangolin) software tool (v3.1.5) (20).

### Phylogenetic and Bayesian evolutionary analyses of P.1 lineage.

The SARS-CoV-2 P.1 sequences and sublineages obtained from this study were subjected to maximum likelihood (ML) phylogenetic analysis using IQ-TREE v2.1.2 (GTR+F+R4, SHaLRT with 1,000 replicates). Subclades, which were formed with more than five sequences or nucleotide substitutions per site ≥ 1 × 10^−4^, were analyzed to characterize their particular mutations.

To perform the Bayesian evolutionary analyses (molecular clock), SARS-CoV-2 P.1 sequences obtained from this study (Data set A; Table S2; *n* = 33) were aligned (using MAFFT v7.475) with 356 high quality sequences belonging to B.1.1.28 and P.1 clades that were available from the EpiCoV database in the GISAID (21). The data set information is detailed in the Supplemental Material.

The maximum likelihood (ML) tree was built with IQ-TREE v2.1.3 (22) under the GTR+F+R3 nucleotide substitution model, as selected by ModelFinder (23). Branch support was calculated using the Shimodaira-Hasegawa (SHaLRT) approximate likelihood ratio test with 1,000 replicates. The temporal signal was assessed by a regression analysis of the root-to-tip genetic distance against sampling dates using TempEst v1.5.3 (24).

Bayesian analysis was performed with the BEAST v2.6.5 (25) software package. The tree was reconstructed using a strict molecular clock model, GTR + F + I + G4 nucleotide substitution model, and a nonparametric Bayesian skyline (BSKL) model (26) as the coalescent tree prior. Five independent runs were performed with 150 million MCMC chains. Convergence and mixing sequences were performed using TRACER v1.7.2. (27). The maximum clade credibility (MCC) tree was summarized with TreeAnnotator v2.6.4. The ancestral character reconstruction (ACR) of epidemic regions was performed with PastML with Marginal Posterior Probabilities Approximation (MPPA) using an F81-like model (28) and visualized using iTol (29).

### Ethical statement.

This study was approved by the Ethics Committees from Hospital de Clínicas de Porto Alegre (CAAE: 30767420.2.0000.5327).

### Data availability.

The sequences were deposited into the GISAID database (https://www.gisaid.org/) (Table S1).
