# Supplementary material for: Genomic Surveillance of SARS-CoV-2 Lineages Indicates Early Circulation of P.1 (Gamma) Variant of Concern in Southern Brazil
Source: Microbiol Spectr. 2022 Feb 16;10(1):e01511-21. doi: 10.1128/spectrum.01511-21 (PMC8849062; doi:10.1128/spectrum.01511-21)
Supplement: SUPPLEMENTAL FILE 1 — Supplemental material. Download SPECTRUM01511-21_Supp_1_seq8.pdf, PDF file, 0.6 MB [file spectrum01511-21_supp_1_seq8.pdf]

Supplemental Material

Methodology

Sampling and RT-qPCR

The specimens were previously submitted to real-time Reverse Transcriptase Polymerase Chain Reaction (RT-qPCR) testing for two genes of the nucleocapsid protein (N1 and N2) for SARS-CoV-2 (1). A total of 202 SARS-CoV-2 specimens (75 from 2020 and 127 from 2021) were included in the study. Eighty-six of them were selected for a Clinical Trial and 116 were selected as a part of a genomic surveillance study in a tertiary-care hospital which is a COVID-19 referral center in Southern Brazil. All specimens included presented a lower cycle threshold (Ct) value (≤25.99) of the RT-qPCR assay.

Next-generation sequencing

Sequencing libraries were prepared using the CleanPlex SARS-CoV-2 panel (Paragon Genomics, Hayward, United States) protocol for target enrichment and library preparation, following manufacturer instructions (https://www.paragongenomics.com/wp-content/uploads/2020/03/UG4001-01_-CleanPlex-SARS-CoV-2-Panel-User-Guide.pdf). The resulting libraries were sequenced in Illumina MiSeq (Illumina, San Diego, US). Consensus sequences were generated by the QIASeq SARS-CoV-2 pipeline (QIAGEN CLC Genomics Workbench 21, Germantown, United States) with high quality whole-genome sequence (<6% Ns, >29.8Kb). The specimens were classified using the Phylogenetic Assignment of Named Global Outbreak Lineages (Pangolin) software tool (v3.1.5) (2) and the sequences were deposited into the GISAID database (Table S1).

Genomic Dataset and Bayesian Analyses

The datasets used in Bayesian analyses are described as follows:

Dataset A) SARS-CoV-2 P.1 sequences (<6% Ns; >29.8 kb) obtained from this study (Table S2; n = 33) collected up to February, 2021;

Dataset B) B.1.1.28 sequences (<1% Ns; >29.8 kb) from Amazonas (n=49) and Rio Grande do Sul (n=173) available on GISAID; collected between July 1, 2020 to February 1, 2021;

Dataset C) P.1 sequences (<1% Ns; >29.8 kb) from Amazonas (n=127) and Rio Grande do Sul (n=4); available on GISAID collected up to February 01, 2021;

Dataset D) Six oldest P.1 sequences (<10% Ns; >29.8 kb) from other Brazilian states, available on GISAID collected until November 30, 2020.

The datasets B, C and D (Table S2) were reclassified by using the Pangolin v3.1.5 software tool (2,3) and nextclade (4). Strains with a different classification than expected or repeated in the dataset were excluded from the next analyses.

After, the sequences were aligned using MAFFT v7.475 (Katoh and Standley 2013) and subjected to maximum likelihood (ML) phylogenetic analysis using IQ-TREE v2.1.3 (5) under the GTR+F+R3 nucleotide substitution model, as selected by the ModelFinder (6) application. Branch support was assessed by the approximate-ratio test based on the Shimodaira–Hasegawa procedure (SHaLRT) with 1000 replicates. The temporal signal was assessed by the regression analysis of the root-to-tip genetic distance against sampling dates using the TempEst v1.5.3 (7).

Bayesian analysis was performed with Bayesian Evolutionary Analysis by Sampling Trees (BEAST) v2.6.5 (8) software package. The tree was reconstructed using a strict molecular clock model, GTR + F + I + G4 nucleotide substitution model, and a non-parametric Bayesian skyline (BSKL) model (9) as the coalescent tree prior. Five independent runs were performed with 150 million MCMC chains. Convergence and mixing samples were performed

using TRACER v1.7.2. (10). The maximum clade credibility (MCC) tree was summarized with TreeAnnotator v2.6.4. All trees built in this study were visualized using FigTree v1.4.4 (http://tree.bio.ed.ac.uk/software/gtree/).The ancestral character reconstruction (ACR) of epidemic regions was performed with PastML with Marginal Posterior Probabilities Approximation (MPPA) with an F81-like model (11) and visualized using iTol (12). The Phylogenetic and Bayesian trees were designed using ggtree v3.0.2 (13), treeio v1.16.1 (14) and ggplot2 (15) on R v4.1.0 (R Core Team, 2020). The map (Figure S1) was designed using geobr (16), dplyr v1.0.6 (17) and ggplot2 (15). Root-to-tip (Figure S2) was performed using exported data from TempEst v1.5.3 (7) and designed using ggplot2 (15).

Supplementary Tables

Table S1. Characteristics of sequenced specimens and setting of specimen recovery

Specimen ID	Date	City of origin	Age Range	Ct N1	Ct N2	Variant	Coverage	GISAID ID (EPI_ISL_)
9_HCPA	Mar-20	Porto Alegre	>65	13.15	11.90	B.1	790.9	1163693
10_HCPA	Mar-20	Porto Alegre	46-55	16.76	15.44	B.1.1.161	1151.3	1163694
11_HCPA	Mar-20	Porto Alegre	46-55	16.95	17.42	B.1.1.161	521.7	1163695
13_HCPA	Apr-20	Porto Alegre	>65	16.66	16.54	B.1.1.161	1018.3	1163696
14_HCPA	Apr-20	Taquari	56-65	17.60	17.80	B.1.1.161	888.4	1163697
15_HCPA	May-20	Porto Alegre	36-45	15.46	14.91	B.1.1.161	1753.7	1163698
16_HCPA	May-20	Charqueadas	56-65	14.77	14.36	B.1.1.161	848.2	1163699
17_HCPA	May-20	Porto Alegre	36-45	18.52	19.30	B.1.1	1271.3	1163700
19_HCPA	Jun-20	Porto Alegre	56-65	13.54	13.22	B.1.1.161	1299.3	1163701
20_HCPA	Jun-20	Esteio	56-65	12.30	12.81	B.1.1.161	1449.6	1163702
22_HCPA	Jul-20	Porto Alegre	56-65	11.17	11.01	B.1.1.409	724.9	1163703
23_HCPA	Jul-20	Canoas	56-65	13.80	13.10	B.1.1.28	981.8	1163704
24_HCPA	Jul-20	Taquara	36-45	11.88	12.34	B.1.1.33	1651.6	1163705
25_HCPA	Aug-20	Butiá	46-55	14.83	15.09	B.1.1.161	1813.1	1163706
26_HCPA	Aug-20	Porto Alegre	56-65	13.00	12.09	B.1.1.161	527.2	1163707
27_HCPA	Aug-20	Faria Lemos	>65	12.89	12.51	B.1.91	712.9	1163708
28_HCPA	Sep-20	Unknown	36-45	13.04	12.49	B.1.1.28	2104.6	1163709
29_HCPA	Sep-20	Guaíba	36-45	14.49	14.02	B.1.1.28	1286.9	1163710
30_HCPA	Sep-20	Porto Alegre	56-55	15.48	15.66	B.1.1.28	1086.1	1163711
31_HCPA	Oct-20	Ijuí	36-45	14.20	13.96	B.1.1.28	1048.8	1163712
33_HCPA	Oct-20	Porto Alegre	26-35	15.40	14.30	B.1.1.161	552.6	1163736
34_HCPA	Nov-20	Canoas	36-45	22.65	24.93	B.1.1.28	578.7	1163713
35_HCPA	Nov-20	Viamão	56-65	11.38	11.32	B.1.1.12	545.6	1163737
37_HCPA	Dec-20	Porto Alegre	36-45	13.82	13.14	P.2	542.3	1163714
38_HCPA	Dec-20	Viamão	36-45	11.50	9.39	B.1.1.28	1194.6	1163715
39_HCPA	Dec-20	Porto Alegre	46-55	12.75	12.55	B.1.1.28	1090.5	1163716
1_LABRESIS	Feb-21	Alvorada	36-45	17.99	19.61	P.1	2341	1133120
2_LABRESIS	Jan-21	Porto Alegre	36-45	13.65	13.41	B.1.1.28	2101	1163530
3_LABRESIS	Feb-21	Porto Alegre	15-25	17.43	19.11	P.1	2073	1133121
4_LABRESIS	Feb-21	Porto Alegre	26-35	11.29	12.62	P.1	1945	1133134
5_LABRESIS	Feb-21	Viamão	26-35	12.73	14.55	P.1	2418	1133123
6_LABRESIS	Feb-21	Porto Alegre	26-35	17.14	17.85	P.1.2	3070	1133124
7_LABRESIS	Feb-21	Porto Alegre	15-25	16.52	17.35	P.1	2627	1133125
8_LABRESIS	Feb-21	Porto Alegre	15-25	12.57	12.82	P.1	2474	1133126
9_LABRESIS	Feb-21	Porto Alegre	36-45	17.91	17.48	P.1	2247	1133127
10_LABRESIS	Feb-21	Porto Alegre	26-35	16.26	16.88	P.1	2352	1133128
11_LABRESIS	Feb-21	Porto Alegre	26-35	14.21	16.33	P.1	2249	1133129
12_LABRESIS	Feb-21	Porto Alegre	56-65	11.79	11.35	P.1	2708	1133130

13_LABRESIS	Feb-21	Porto Alegre	<3	19.90	19.97	P.1	2403	1133131
14_LABRESIS	Feb-21	Porto Alegre	<3	11.42	11.08	P.1	1527	1133132
15_LABRESIS	Jan-21	Porto Alegre	<3	14.37	13.24	B.1.1.28	2367	1161401
16_LABRESIS	Jan-21	Gravataí	26-35	12.49	11.99	P.1	2763	1133133
17_LABRESIS	Jan-21	Porto Alegre	26-35	17.01	17.31	P.2	2331	1161402
18_LABRESIS	Jan-21	Porto Alegre	26-35	10.98	9.80	P.2	2674	1161403
19_LABRESIS	Jan-21	Gravataí	<3	15.04	15.48	P.2	2252	1161404
20_LABRESIS	Jan-21	Porto Alegre	26-35	16.07	15.91	B.1.575	2316	1161405
21_LABRESIS	Jan-21	Tramandaí	26-35	12.11	12.03	P.2	2268	1161406
22_LABRESIS	Jan-21	Porto Alegre	26-35	15.52	12.50	P.2	1964	1161407
23_LABRESIS	Jan-21	Porto Alegre	26-35	15.26	14.15	P.2	2459	1161408
24_LABRESIS	Jan-21	Gravataí	26-35	15.94	15.10	P.2	1824	1161409
25_LABRESIS	Jan-21	Porto Alegre	<3	17.30	16.80	P.2	2059	1163532
26_LABRESIS	Feb-21	Porto Alegre	26-35	13.44	11.31	P.1	2354	1133134
27_LABRESIS	Jan-21	Porto Alegre	<3	19.15	19.32	B.1.1.28	2402	1161410
28_LABRESIS	Jan-21	Porto Alegre	56-65	14.89	15.28	P.2	2517	1161411
30_LABRESIS	Jan-21	Porto Alegre	<3	14.86	15.17	B.1.1.28	1822	1161412
31_LABRESIS	Feb-21	Porto Alegre	26-35	16.17	15.67	P.1	2369	1133135
32_LABRESIS	Feb-21	Porto Alegre	56-65	18.24	19.23	B.1.1.28	2691	1161413
33_LABRESIS	Feb-21	Porto Alegre	56-65	21.67	22.29	B.1.1.161	1494	1161414
34_LABRESIS	Feb-21	Porto Alegre	46-55	10.81	10.38	P.1	1729	1133136
35_LABRESIS	Feb-21	Porto Alegre	18-25	11.56	13.14	P.1	2776	1133137
36_LABRESIS	Feb-21	Porto Alegre	46-55	17.90	18.20	P.1	2499	1133138
37_LABRESIS	Feb-21	Porto Alegre	26-35	16.96	16.60	P.1	6764	1133139
39_LABRESIS	Feb-21	Porto Alegre	36-45	16.10	17.50	P.1	2047	1133140
40_LABRESIS	Feb-21	Porto Alegre	26-35	12.03	12.41	B.1.1.28	1977	1161415
41_LABRESIS	Feb-21	Porto Alegre	26-35	21.77	24.62	P.1	2394	1133141
42_LABRESIS	Feb-21	Porto Alegre	46-55	12.67	12.51	P.1	2180	1133142
43_LABRESIS	Feb-21	Triunfo	26-35	10.13	11.40	P.1	2128	1133143
44_LABRESIS	Feb-21	Porto Alegre	36-45	17.70	18.90	P.1	1880	1133144
69_LABRESIS	Jan-21	Barão	56-65	19.17	24.38	P.2	2063.6	2098666
70_LABRESIS	Feb-21	Porto Alegre	46-55	20.53	20.27	P.1	1780.6	2086585
71_LABRESIS	Feb-21	Porto Alegre	56-65	18.85	19.45	P.2	2101.9	2086586
72_LABRESIS	Feb-21	Porto Alegre	46-55	14.75	15.35	P.1	1781.4	2086587
73_LABRESIS	Feb-21	Guaíba	15-25	17.68	18.58	P.1	1872.3	2086588
74_LABRESIS	Feb-21	Porto Alegre	15-25	19.37	22.23	P.1	1160.5	2086589
75_LABRESIS	Feb-21	Porto Alegre	56-65	24.83	25.87	P.1	353.5	2086763
76_LABRESIS	Feb-21	Porto Alegre	15-25	21.13	21.27	P.1.1	2058.2	2086590
77_LABRESIS	Feb-21	Gravataí	56-65	19.39	19.15	P.1.1	624.5	2086764
78_LABRESIS	Feb-21	Canoas	26-35	20.28	20.14	P.1	1756.5	2086591
79_LABRESIS	Feb-21	Porto Alegre	46-55	19.37	19.38	P.1	1493.3	2086592
80_LABRESIS	Feb-21	Porto Alegre	36-45	12.80	19.15	P.1	2001.7	2086593
81_LABRESIS	Mar-21	Porto Alegre	56-65	17.33	18.43	P.1	1649.7	2086594
82_LABRESIS	Mar-21	Porto Alegre	26-35	18.99	19.59	P.1	1894.8	2086595
83_LABRESIS	Mar-21	Viamão	46-55	17.80	18.02	P.1	2723.5	2086596
84_LABRESIS	Mar-21	Alvorada	56-65	20.29	20.20	P.1	2130.4	2086597
85_LABRESIS	Mar-21	Viamão	46-55	21.39	21.06	P.1	1948.7	2086598
86_LABRESIS	Mar-21	Porto Alegre	15-25	18.24	19.66	P.1	1943.7	2086599
87_LABRESIS	Mar-21	Porto Alegre	46-55	19.76	20.79	P.1	1780.9	2086600
88_LABRESIS	Mar-21	Chuvisca	36-45	19.00	19.75	P.2	2113.6	2086601
89_LABRESIS	Mar-21	Porto Alegre	56-65	20.08	20.87	P.1	2900.1	2086602
90_LABRESIS	Mar-21	Porto Alegre	46-55	25.12	25.34	P.1.2	1276.1	2086603

91_LABRESIS	Mar-21	Porto Alegre	56-65	15.00	16.10	P.1	2231	2086604
92_LABRESIS	Mar-21	Porto Alegre	36-45	15.19	14.28	P.1	1972.5	2086605
93_LABRESIS	Mar-21	Porto Alegre	26-35	23.73	23.59	P.1	1149.1	2086606
94_LABRESIS	Mar-21	Parobé	46-55	14.30	15.60	P.1	1864.3	2086607
96_LABRESIS	Mar-21	Porto Alegre	56-65	24.17	25.99	P.1	1153.7	2086608
98_LABRESIS	Mar-21	Porto Alegre	46-55	15.38	15.12	P.1	1329.2	2086609
100_LABRESIS	Apr-21	Porto Alegre	56-65	21.74	21.24	P.1	1201.1	2086610
101_LABRESIS	Apr-21	Porto Alegre	46-55	20.48	20.11	P.1.2	1689.4	2086611
102_LABRESIS	Apr-21	Porto Alegre	46-55	18.08	19.39	P.1	1815.1	2086612
103_LABRESIS	Apr-21	Alvorada	56-65	23.06	23.03	P.1	634.3	2086765
104_LABRESIS	Apr-21	Porto Alegre	36-45	15.19	19.45	P.1	2141.8	2086613
105_LABRESIS	Apr-21	Porto Alegre	26-35	15.73	15.52	P.1	2981.6	2086614
106_LABRESIS	Apr-21	Porto Alegre	56-65	20.04	20.03	P.1	1697.7	2086615
107_LABRESIS	Apr-21	Porto Alegre	46-55	22.79	22.98	P.1	1695	2086616
108_LABRESIS	Apr-21	Porto Alegre	26-35	15.10	15.20	P.1	1984.3	2086617
109_LABRESIS	Apr-21	Porto Alegre	56-65	14.98	16.35	P.1	2061.5	2086618
110_LABRESIS	Apr-21	Porto Alegre	36-45	19.74	18.55	P.1	1895.7	2086619
112_LABRESIS	Apr-21	Porto Alegre	56-65	22.94	24.79	P.1	2040.3	2086620
113_LABRESIS	Apr-21	Porto Alegre	46-55	17.12	16.58	P.1	2620.8	2086621
114_LABRESIS	Apr-21	Alvorada	56-65	23.33	22.85	P.1	3517	2086622
116_LABRESIS	Apr-21	Canoas	56-65	22.54	21.81	P.1	2967.5	2086767
117_LABRESIS	Jun-20	Porto Alegre	36-45	17.49	18.35	B.1.1.161	1747.3	3218208
118_LABRESIS	Jun-20	Porto Alegre	46-55	18.81	20.44	B.1.1.161	1971.8	3218210
119_LABRESIS	Jun-20	Porto Alegre	46-55	21.36	21.93	B.1.91	1656.4	3218212
121_LABRESIS	Jun-20	Porto Alegre	36-45	20.41	21.35	B.1.1.28	1011.7	3218215
122_LABRESIS	Jun-20	Porto Alegre	56-65	21.18	21.63	B.1.91	1690.1	3218217
123_LABRESIS	Jun-20	Gravataí	46-55	21.90	22.09	B.1.1.28	1643.3	3218219
124_LABRESIS	Jun-20	Porto Alegre	56-65	23.87	23.85	B.1.1.161	1287.2	3218221
125_LABRESIS	Jul-20	Porto Alegre	26-35	20.94	22.59	B.1.1.28	1424.4	3218222
126_LABRESIS	Jul-20	Porto Alegre	56-65	14.17	17.61	B.1.91	2301.9	3218224
127_LABRESIS	Jul-20	Barra do Ribeiro	56-65	14.56	14.49	B.1.1.161	1763.2	3218226
128_LABRESIS	Jul-20	Porto Alegre	56-65	15.67	16.32	B.1.1.161	1791.1	3218228
129_LABRESIS	Jul-20	Porto Alegre	18-25	18.47	18.70	B.1.1	1671.5	3218229
130_LABRESIS	Aug-20	Porto Alegre	26-35	13.37	13.60	B.1.161	1980.2	3218231
131_LABRESIS	Aug-20	Camaquã	56-65	22.43	22.71	B.1.28	1063.7	3218233
133_LABRESIS	Aug-20	Porto Alegre	56-65	21.51	21.87	B.1.28	1453.6	3218235
134_LABRESIS	Aug-20	Gravataí	56-65	19.96	19.30	B.1.1.409	1996.8	3218237
135_LABRESIS	Aug-20	Porto Alegre	46-55	22.07	21.29	B.1.1.462	1672.7	3218238
136_LABRESIS	Aug-20	Porto Alegre	56-65	23.27	23.50	B.1.1.28	1761.5	3218240
137_LABRESIS	Aug-20	Canoas	56-65	23.64	23.23	B.1.1.161	1679.8	3218242
138_LABRESIS	Aug-20	Porto Alegre	56-65	21.14	20.33	B.1.1.161	2270.8	3218244
139_LABRESIS	Aug-20	Gravataí	36-45	19.70	19.08	B.1.1.161	2707.8	3218245
141_LABRESIS	Aug-20	Portão	46-55	22.83	23.90	B.1.1.33	879.1	3218247
142_LABRESIS	Aug-20	Viamão	46-55	11.27	11.73	B.1.1.161	2818.6	3218249
143_LABRESIS	Sep-20	Porto Alegre	46-55	24.54	25.00	B.1.1	1275.4	3218251
144_LABRESIS	Sep-20	Canoas	56-65	20.38	21.04	B.1.1.161	1484	3218253
145_LABRESIS	Sep-20	Porto Alegre	56-65	22.69	21.93	B.1.1.28	1279.7	3218254
146_LABRESIS	Sep-20	Eldorado do Sul	46-55	20.22	20.09	B.1.1.28	1385.1	3218256
147_LABRESIS	Sep-20	Porto Alegre	36-45	23.3	23.97	B.1.1.28	912.3	3218258
148_LABRESIS	Oct-20	Porto Alegre	36-45	25.69	18.12	B.1.1.161	1772.6	3218259
149_LABRESIS	Oct-20	Porto Alegre	36-45	20.64	18.90	B.1.1.33	1996.1	3233183
150_LABRESIS	Oct-20	Porto Alegre	36-45	21.36	20.12	B.1.1.370	2628	3218261

151_LABRESIS	Oct-20	Novo Hamburgo	56-65	18.09	19.22	B.1.1.161	2358	3218263
152_LABRESIS	Oct-20	Porto Alegre	18-25	24.3	24.39	B.1.1	1684.4	3218265
153_LABRESIS	Oct-20	Porto Alegre	56-65	19.24	18.45	B.1.1	2812	3218266
154_LABRESIS	Oct-20	Portão	56-65	24.10	25.60	B.1.1.33	1610.9	3218268
157_LABRESIS	Nov-20	Porto Alegre	46-55	22.31	22.71	B.1.1.28	1656.8	3218270
158_LABRESIS	Nov-20	Porto Alegre	56-65	17.69	16.81	B.1.1.28	2550.6	3218271
159_LABRESIS	Nov-20	Alvorada	56-65	23.99	22.86	P.2	1915.4	3218273
160_LABRESIS	Nov-20	Porto Alegre	46-55	22.37	22.53	B.1.1.28	1925.4	3218275
161_LABRESIS	Nov-20	Cachoeirinha	46-55	20.64	19.68	B.1.1.28	3103.1	3218277
162_LABRESIS	Nov-20	Porto Alegre	56-65	17.42	17.13	B.1.1.28	4792.9	3218278
163_LABRESIS	Nov-20	Taquara	56-65	18.86	18.71	B.1.1	2824.4	3218280
164_LABRESIS	Nov-20	Alvorada	46-55	21.41	22.33	P.1	1440.9	3233232
165_LABRESIS	Mar-20	Porto Alegre	56-65	22.17	25.94	B.1.1.28	3164.1	2617875
166_LABRESIS	Aug-20	Porto Alegre	56-65	25.07	24.49	B.1.1.28	1767.2	2617876
167_LABRESIS	Nov-20	Palmares do Sul	46-55	19.32	20.34	P.2	3106.1	2617877
169_LABRESIS	Dec-20	Porto Alegre	15-25	17.17	15.83	B.1.1.161	3542.6	2617878
170_LABRESIS	Dec-20	Viamão	26-32	25.11	24.18	B.1.1.28	2230.2	2617879
171_LABRESIS	May-21	Porto Alegre	36-45	11.93	8.88	P.1	2852.9	2617880
172_LABRESIS	May-21	Porto Alegre	46-55	19.21	18.44	P.1	3489.5	2617881
173_LABRESIS	May-21	Porto Alegre	46-55	24.77	23.79	P.1	536.3	2617882
175_LABRESIS	May-21	Porto Alegre	56-65	18.67	19.99	P.1	1628	2617883
176_LABRESIS	May-21	Viamão	26-35	14.51	14.70	P.1	2068.6	2617884
177_LABRESIS	May-21	Porto Alegre	26-35	18.10	14.54	P.1	2097.1	2617885
178_LABRESIS	May-21	Porto Alegre	56-65	21.14	19.94	P.1	1704.1	2617886
179_LABRESIS	May-21	Canoas	26-35	17.12	16.74	P.1	1646.6	2617887
180_LABRESIS	May-21	Porto Alegre	36-45	13.15	12.75	P.1	2159.3	2617888
181_LABRESIS	May-21	Canoas	36-45	13.48	14.15	P.1	2418.4	2617889
182_LABRESIS	May-21	Porto Alegre	36-45	14.35	14.51	P.1	2679.9	2617890
183_LABRESIS	May-21	Porto Alegre	56-65	17.94	17.80	P.1	2583.8	2617891
184_LABRESIS	May-21	Nova Petrópolis	36-45	15.46	16.25	P.1	3310.3	2617892
185_LABRESIS	May-21	Porto Alegre	36-45	12.27	13.00	P.1	2751.8	2617893
186_LABRESIS	May-21	Canoas	46-55	15.13	14.55	P.1	2903.6	2617894
187_LABRESIS	May-21	Panambi	46-55	12.92	12.10	P.1	2459.5	2617895
188_LABRESIS	May-21	Porto Alegre	46-55	13.52	12.95	P.1	2887.1	2617896
189_LABRESIS	May-21	Porto Alegre	26-35	10.70	10.90	P.1	2237.9	2617897
190_LABRESIS	May-21	Porto Alegre	46-55	12.90	19.90	P.1	2221.3	2617898
191_LABRESIS	May-21	Sapucaia do Sul	56-65	10.20	9.00	P.1	1783.7	2617899
192_LABRESIS	May-21	Porto Alegre	56-65	18.90	19.00	P.1	2194.1	2617900
193_LABRESIS	May-21	Porto Alegre	56-65	15.43	13.92	P.1	2126.1	2617901
194_LABRESIS	May-21	Porto Alegre	46-55	15.14	14.84	P.1	3254.7	2617902
195_LABRESIS	May-21	Porto Alegre	26-35	16.33	14.40	P.1.2	1904.2	2617903
196_LABRESIS	May-21	Porto Alegre	36-45	14.19	14.41	P.1	2135.9	2617904
197_LABRESIS	May-21	Porto Alegre	36-45	14.77	15.14	P.1	3047.5	2617905
198_LABRESIS	May-21	Porto Alegre	46-55	16.92	16.52	P.1.2	2965.5	2617906
199_LABRESIS	May-21	Santa Maria	36-45	18.71	17.65	P.1	2905	2617907
200_LABRESIS	May-21	Canoas	26-35	16.67	15.28	P.1	3038	2617908
201_LABRESIS	May-21	São Lourenço do Sul	46-55	14.44	15.24	P.1	3347.6	2617909
202_LABRESIS	May-21	Canoas	26-35	13.18	13.85	P.1	3768.4	2617910
203_LABRESIS	May-21	Itaqui	15-25	16.48	16.56	C.37	3142.8	2617911
204_LABRESIS	May-21	Porto Alegre	36-45	18.18	18.48	P.1	3237.9	2617912
205_LABRESIS	May-21	Porto Alegre	26-35	21.87	22.36	P.1	2970.8	2617913
206_LABRESIS	May-21	Canoas	15-25	11.54	10.83	P.1	2943.3	2617914
207_LABRESIS	May-21	Sapiranga	46-55	19.42	18.74	P.1	2531.7	2617915
208_LABRESIS	May-21	Porto Alegre	46-55	13.00	15.93	P.1	3010.2	2617916

209_LABRESIS	May-21	Porto Alegre	46-55	11.15	10.47	P.1	3509.1	2617917
210_LABRESIS	May-21	Ibirubá	26-35	19.72	19.02	P.1	2909.5	2617918
211_LABRESIS	May-21	Viamão	56-65	18.31	17.78	P.1.2	2567.6	2617919
212_LABRESIS	May-21	Porto Alegre	36-45	13.47	12.75	P.1	2507.8	2617920

*Cycle threshold (Ct) values obtained by RT-qPCR assay using the two genes of the nucleocapsid protein, N1 and N2, as targets according to CDC/USA protocol.

Table S2. Dataset sequences A, B, C and D used for Bayesian analysis.

Dataset A	EPI_ISL_1133120	hCoV-19/Brazil/RS-1RCSHCPA/2021
	EPI_ISL_1133121	hCoV-19/Brazil/RS-3BPCHCPA/2021
	EPI_ISL_1133122	hCoV-19/Brazil/RS-4BOGHCPA/2021
	EPI_ISL_1133123	hCoV-19/Brazil/RS-5DSTHCPA/2021
	EPI_ISL_1133125	hCoV-19/Brazil/RS-7RMSGHCPA/2021
	EPI_ISL_1133126	hCoV-19/Brazil/RS-8IKRSHCPA/2021
	EPI_ISL_1133127	hCoV-19/Brazil/RS-9JMMHCPA/2021
	EPI_ISL_1133128	hCoV-19/Brazil/RS-10EFMHCPA/2021
	EPI_ISL_1133129	hCoV-19/Brazil/RS-11AGHCPA/2021
	EPI_ISL_1133130	hCoV-19/Brazil/RS-12ACAHCPA/2021
	EPI_ISL_1133131	hCoV-19/Brazil/RS-13VVBVHCPA/2021
	EPI_ISL_1133132	hCoV-19/Brazil/RS-14MBMHCPA/2021
	EPI_ISL_1133133	hCoV-19/Brazil/RS-16JMHCPA/2021
	EPI_ISL_1133134	hCoV-19/Brazil/RS-26MRHCPA/2021
	EPI_ISL_1133135	hCoV-19/Brazil/RS-31AWDHCPA/2021
	EPI_ISL_1133136	hCoV-19/Brazil/RS-34VAMHCPA/2021
	EPI_ISL_1133137	hCoV-19/Brazil/RS-35ASMHCPA/2021
	EPI_ISL_1133138	hCoV-19/Brazil/RS-36FPPHCPA/2021
	EPI_ISL_1133139	hCoV-19/Brazil/RS-37MMCHCPA/2021
	EPI_ISL_1133140	hCoV-19/Brazil/RS-39SMHHCPA/2021
	EPI_ISL_1133141	hCoV-19/Brazil/RS-41LNPLHCPA/2021
	EPI_ISL_1133142	hCoV-19/Brazil/RS-42JBAHCPA/2021
	EPI_ISL_1133143	hCoV-19/Brazil/RS-43RPAMHCPA/2021
	EPI_ISL_1133144	hCoV-19/Brazil/RS-44JSLHCPA/2021
	EPI_ISL_2086585	hCoV-19/Brazil/RS-70_LABRESIS/2021
	EPI_ISL_2086587	hCoV-19/Brazil/RS-72_LABRESIS/2021
	EPI_ISL_2086588	hCoV-19/Brazil/RS-73_LABRESIS/2021
	EPI_ISL_2086589	hCoV-19/Brazil/RS-74_LABRESIS/2021
	EPI_ISL_2086591	hCoV-19/Brazil/RS-78_LABRESIS/2021
	EPI_ISL_2086592	hCoV-19/Brazil/RS-79_LABRESIS/2021

	EPI_ISL_2086593	hCoV-19/Brazil/RS-80_LABRESIS/2021
	EPI_ISL_2086763	hCoV-19/Brazil/RS-LABRESIS-75/2021
	EPI_ISL_3233232	hCoV-19/Brazil/RS-164_LABRESIS/2020
Dataset B	EPI_ISL_1068089	hCoV-19/Brazil/AM-FIOCRUZ-20141355VB/2020
	EPI_ISL_1068090	hCoV-19/Brazil/AM-FIOCRUZ-20141399MC/2020
	EPI_ISL_1068093	hCoV-19/Brazil/AM-FIOCRUZ-20141562YS/2020
	EPI_ISL_1068095	hCoV-19/Brazil/AM-FIOCRUZ-20141671CB/2020
	EPI_ISL_1068096	hCoV-19/Brazil/AM-FIOCRUZ-20141721RM/2020
	EPI_ISL_1068101	hCoV-19/Brazil/AM-FIOCRUZ-20142153MO/2020
	EPI_ISL_1068102	hCoV-19/Brazil/AM-FIOCRUZ-20142189LL/2020
	EPI_ISL_1068107	hCoV-19/Brazil/AM-FIOCRUZ-20142424SS/2020
	EPI_ISL_1068127	hCoV-19/Brazil/AM-FIOCRUZ-20842451CN/2020
	EPI_ISL_1068132	hCoV-19/Brazil/AM-FIOCRUZ-20842572LS/2020
	EPI_ISL_1068134	hCoV-19/Brazil/AM-FIOCRUZ-20842652AF/2020
	EPI_ISL_1068135	hCoV-19/Brazil/AM-FIOCRUZ-20842657BJ/2020
	EPI_ISL_1068137	hCoV-19/Brazil/AM-FIOCRUZ-20842695EA/2020
	EPI_ISL_1068146	hCoV-19/Brazil/AM-FIOCRUZ-20842986CO/2020
	EPI_ISL_1068148	hCoV-19/Brazil/AM-FIOCRUZ-20843023JA/2020
	EPI_ISL_1068152	hCoV-19/Brazil/AM-FIOCRUZ-20843242IB/2020
	EPI_ISL_1068161	hCoV-19/Brazil/AM-FIOCRUZ-20843383RG/2020
	EPI_ISL_1068190	hCoV-19/Brazil/AM-FIOCRUZ-20890297AX/2020
	EPI_ISL_1068192	hCoV-19/Brazil/AM-FIOCRUZ-20890511ME/2020
	EPI_ISL_1068205	hCoV-19/Brazil/AM-FIOCRUZ-20891452AC/2020
	EPI_ISL_1068206	hCoV-19/Brazil/AM-FIOCRUZ-20891505PT/2020
	EPI_ISL_1068208	hCoV-19/Brazil/AM-FIOCRUZ-20891515LA/2020
	EPI_ISL_1068209	hCoV-19/Brazil/AM-FIOCRUZ-20891550FA/2020
	EPI_ISL_1068223	hCoV-19/Brazil/AM-FIOCRUZ-20892224RM/2020
	EPI_ISL_1068253	hCoV-19/Brazil/AM-FIOCRUZ-20895570KS/2020
	EPI_ISL_1068254	hCoV-19/Brazil/AM-FIOCRUZ-20895654ID/2020
	EPI_ISL_1068257	hCoV-19/Brazil/AM-FIOCRUZ-20898137RM/2020
	EPI_ISL_1661251	hCoV-19/Brazil/AM-FIOCRUZ-20842519JC/2020
	EPI_ISL_2298865	hCoV-19/Brazil/AM-IEC1200-178756/2020
	EPI_ISL_801386	hCoV-19/Brazil/AM-FIOCRUZ-20141882LT/2020
	EPI_ISL_801387	hCoV-19/Brazil/AM-FIOCRUZ-20141937RS/2020
	EPI_ISL_801388	hCoV-19/Brazil/AM-FIOCRUZ-20141978DR/2020

	EPI_ISL_801389	hCoV-19/Brazil/AM-FIOCRUZ-20142116AS/2020
	EPI_ISL_801390	hCoV-19/Brazil/AM-FIOCRUZ-20142279MP/2020
	EPI_ISL_801391	hCoV-19/Brazil/AM-FIOCRUZ-20142492MG/2020
	EPI_ISL_801392	hCoV-19/Brazil/AM-FIOCRUZ-20142493RO/2020
	EPI_ISL_801393	hCoV-19/Brazil/AM-FIOCRUZ-20842350OC/2020
	EPI_ISL_801394	hCoV-19/Brazil/AM-FIOCRUZ-20842378LB/2020
	EPI_ISL_801395	hCoV-19/Brazil/AM-FIOCRUZ-20842393GA/2020
	EPI_ISL_801396	hCoV-19/Brazil/AM-FIOCRUZ-20842568SB/2020
	EPI_ISL_801397	hCoV-19/Brazil/AM-FIOCRUZ-20890012JJ/2020
	EPI_ISL_801398	hCoV-19/Brazil/AM-FIOCRUZ-20890013MB/2020
	EPI_ISL_801400	hCoV-19/Brazil/AM-FIOCRUZ-20890114ED/2020
	EPI_ISL_801401	hCoV-19/Brazil/AM-FIOCRUZ-20890117JP/2020
	EPI_ISL_833131	hCoV-19/Brazil/AM-FIOCRUZ-20142761FC/2020
	EPI_ISL_833168	hCoV-19/Brazil/AM-988/2020
	EPI_ISL_918512	hCoV-19/Brazil/AM-IEC-177300/2020
	EPI_ISL_925916	hCoV-19/Brazil/AM-IEC-177299/2020
	EPI_ISL_926446	hCoV-19/Brazil/AM-IEC-177302/2020
	EPI_ISL_1161401	hCoV-19/Brazil/RS-15RSLHCPA/2021
	EPI_ISL_1161410	hCoV-19/Brazil/RS-27HRAFHCPA/2021
	EPI_ISL_1161412	hCoV-19/Brazil/RS-30DGSHCPA/2021
	EPI_ISL_1161413	hCoV-19/Brazil/RS-32MBSHCPA/2021
	EPI_ISL_1161415	hCoV-19/Brazil/RS-40FCZVHCPA/2021
	EPI_ISL_1163530	hCoV-19/Brazil/2PWLHCPA/2021
	EPI_ISL_1163709	hCoV-19/Brazil/RS-28DBCHCPA/2020
	EPI_ISL_1163710	hCoV-19/Brazil/RS-29VDCHCPA/2020
	EPI_ISL_1163711	hCoV-19/Brazil/RS-30HGGHCPA/2020
	EPI_ISL_1163713	hCoV-19/Brazil/RS-34JVSCHCPA/2020
	EPI_ISL_1163715	hCoV-19/Brazil/RS-38TCBHCPA/2020
	EPI_ISL_1163716	hCoV-19/Brazil/RS-39BRMHCPA/2020
	EPI_ISL_1163736	hCoV-19/Brazil/31GGPHCPA/2020
	EPI_ISL_1182599	hCoV-19/Brazil/RS-FUNED-936440/2020
	EPI_ISL_1182610	hCoV-19/Brazil/RS-FUNED-904220/2020
	EPI_ISL_1182621	hCoV-19/Brazil/RS-FUNED-828330/2020
	EPI_ISL_1182623	hCoV-19/Brazil/RS-FUNED-831330/2020
	EPI_ISL_1195275	hCoV-19/Brazil/RS-34614CVL/2020

	EPI_ISL_1195276	hCoV-19/Brazil/RS-34618CVL/2020
	EPI_ISL_1195277	hCoV-19/Brazil/RS-30960CVL/2020
	EPI_ISL_1195278	hCoV-19/Brazil/RS-29445CVL/2020
	EPI_ISL_1195279	hCoV-19/Brazil/RS-26873CVL/2020
	EPI_ISL_1195280	hCoV-19/Brazil/RS-27045CVL/2020
	EPI_ISL_1195281	hCoV-19/Brazil/RS-34562CVL/2020
	EPI_ISL_1195282	hCoV-19/Brazil/RS-34490CVL/2020
	EPI_ISL_1195283	hCoV-19/Brazil/RS-34738CVL/2020
	EPI_ISL_1195284	hCoV-19/Brazil/RS-33998CVL/2020
	EPI_ISL_1195285	hCoV-19/Brazil/RS-34366CVL/2020
	EPI_ISL_1195286	hCoV-19/Brazil/RS-34379CVL/2020
	EPI_ISL_1195287	hCoV-19/Brazil/RS-31430CVL/2020
	EPI_ISL_1195288	hCoV-19/Brazil/RS-30556CVL/2020
	EPI_ISL_1195289	hCoV-19/Brazil/RS-30175CVL/2020
	EPI_ISL_1195290	hCoV-19/Brazil/RS-28433CVL/2020
	EPI_ISL_1195291	hCoV-19/Brazil/RS-28044CVL/2020
	EPI_ISL_1195292	hCoV-19/Brazil/RS-27081CVL/2020
	EPI_ISL_1195293	hCoV-19/Brazil/RS-22325CVL/2020
	EPI_ISL_1469555	hCoV-19/Brazil/RS-29850CVL/2020
	EPI_ISL_1469560	hCoV-19/Brazil/RS-11684CVL/2020
	EPI_ISL_1469572	hCoV-19/Brazil/RS-19368CVL/2020
	EPI_ISL_1469573	hCoV-19/Brazil/RS-24944CVL/2020
	EPI_ISL_1469576	hCoV-19/Brazil/RS-13343CVL/2020
	EPI_ISL_1469579	hCoV-19/Brazil/RS-19218CVL/2020
	EPI_ISL_1469584	hCoV-19/Brazil/RS-20017CVL/2020
	EPI_ISL_1469586	hCoV-19/Brazil/RS-26231CVL/2020
	EPI_ISL_1469593	hCoV-19/Brazil/RS-15883CVL/2020
	EPI_ISL_1469604	hCoV-19/Brazil/RS-21234CVL/2020
	EPI_ISL_1469608	hCoV-19/Brazil/RS-34418CVL/2020
	EPI_ISL_1469609	hCoV-19/Brazil/RS-11645CVL/2020
	EPI_ISL_1469615	hCoV-19/Brazil/RS-10594CVL/2020
	EPI_ISL_1469620	hCoV-19/Brazil/RS-18377CVL/2020
	EPI_ISL_1469623	hCoV-19/Brazil/RS-13468CVL/2020
	EPI_ISL_1469624	hCoV-19/Brazil/RS-23278CVL/2020
	EPI_ISL_1469625	hCoV-19/Brazil/RS-8698CVL/2020

	EPI_ISL_1469629	hCoV-19/Brazil/RS-19014CVL/2020
	EPI_ISL_1469631	hCoV-19/Brazil/RS-21098CVL/2020
	EPI_ISL_1469633	hCoV-19/Brazil/RS-19973CVL/2020
	EPI_ISL_1469636	hCoV-19/Brazil/RS-34416CVL/2020
	EPI_ISL_1469637	hCoV-19/Brazil/RS-23727CVL/2020
	EPI_ISL_1469638	hCoV-19/Brazil/RS-30570CVL/2020
	EPI_ISL_1469641	hCoV-19/Brazil/RS-34405CVL/2020
	EPI_ISL_1469642	hCoV-19/Brazil/RS-24316CVL/2020
	EPI_ISL_1469647	hCoV-19/Brazil/RS-13263CVL/2020
	EPI_ISL_1469656	hCoV-19/Brazil/RS-5587CVL/2020
	EPI_ISL_1469657	hCoV-19/Brazil/RS-7892CVL/2020
	EPI_ISL_1469658	hCoV-19/Brazil/RS-31603CVL/2020
	EPI_ISL_1469661	hCoV-19/Brazil/RS-21812CVL/2020
	EPI_ISL_1469669	hCoV-19/Brazil/RS-13420CVL/2020
	EPI_ISL_1469675	hCoV-19/Brazil/RS-23455CVL/2020
	EPI_ISL_1469677	hCoV-19/Brazil/RS-22763CVL/2020
	EPI_ISL_1469683	hCoV-19/Brazil/RS-23365CVL/2020
	EPI_ISL_1469684	hCoV-19/Brazil/RS-18354CVL/2020
	EPI_ISL_1469687	hCoV-19/Brazil/RS-21331CVL/2020
	EPI_ISL_1469692	hCoV-19/Brazil/RS-18743CVL/2020
	EPI_ISL_1469696	hCoV-19/Brazil/RS-15273CVL/2020
	EPI_ISL_1469704	hCoV-19/Brazil/RS-30366CVL/2020
	EPI_ISL_1469713	hCoV-19/Brazil/RS-15455CVL/2020
	EPI_ISL_1469714	hCoV-19/Brazil/RS-16305CVL/2020
	EPI_ISL_1469719	hCoV-19/Brazil/RS-30252CVL/2020
	EPI_ISL_1469720	hCoV-19/Brazil/RS-27333CVL/2020
	EPI_ISL_1469721	hCoV-19/Brazil/RS-26493CVL/2020
	EPI_ISL_1469722	hCoV-19/Brazil/RS-25734CVL/2020
	EPI_ISL_1469723	hCoV-19/Brazil/RS-28198CVL/2020
	EPI_ISL_1469730	hCoV-19/Brazil/RS-25360CVL/2020
	EPI_ISL_1469731	hCoV-19/Brazil/RS-18191CVL/2020
	EPI_ISL_1469733	hCoV-19/Brazil/RS-25202CVL/2020
	EPI_ISL_1469737	hCoV-19/Brazil/RS-34631CVL/2020
	EPI_ISL_1469740	hCoV-19/Brazil/RS-29517CVL/2020
	EPI_ISL_1469746	hCoV-19/Brazil/RS-34589CVL/2020

	EPI_ISL_1469748	hCoV-19/Brazil/RS-4772CVL/2020
	EPI_ISL_1469749	hCoV-19/Brazil/RS-15968CVL/2020
	EPI_ISL_1469754	hCoV-19/Brazil/RS-19441CVL/2020
	EPI_ISL_1469774	hCoV-19/Brazil/RS-31118CVL/2020
	EPI_ISL_1469778	hCoV-19/Brazil/RS-16736CVL/2020
	EPI_ISL_1469781	hCoV-19/Brazil/RS-21394CVL/2020
	EPI_ISL_1469783	hCoV-19/Brazil/RS-15744CVL/2020
	EPI_ISL_1469796	hCoV-19/Brazil/RS-5596CVL/2020
	EPI_ISL_1469802	hCoV-19/Brazil/RS-22236CVL/2020
	EPI_ISL_1469803	hCoV-19/Brazil/RS-16090CVL/2020
	EPI_ISL_1469835	hCoV-19/Brazil/RS-11625CVL/2020
	EPI_ISL_1469845	hCoV-19/Brazil/RS-13095CVL/2020
	EPI_ISL_1479121	hCoV-19/Brazil/RS-26120CVL/2020
	EPI_ISL_1799499	hCoV-19/Brazil/RS-00310HM_LMM52636/2020
	EPI_ISL_1799505	hCoV-19/Brazil/RS-00617HM_LMM52648/2020
	EPI_ISL_2249348	hCoV-19/Brazil/RS-FUNED-104235-21/2020
	EPI_ISL_2249352	hCoV-19/Brazil/RS-FUNED-98672-21/2020
	EPI_ISL_2249353	hCoV-19/Brazil/RS-FUNED-96918-21/2020
	EPI_ISL_2249355	hCoV-19/Brazil/RS-FUNED-401-21/2021
	EPI_ISL_2249379	hCoV-19/Brazil/RS-FUNED-2858-21/2021
	EPI_ISL_2249382	hCoV-19/Brazil/RS-FUNED-1120-21/2021
	EPI_ISL_2344429	hCoV-19/Brazil/RS-L111-CD4046/2020
	EPI_ISL_2344433	hCoV-19/Brazil/RS-L94-CD3012/2020
	EPI_ISL_2344454	hCoV-19/Brazil/RS-L101-CD4037/2020
	EPI_ISL_2431429	hCoV-19/Brazil/RS-LMM45505/2021
	EPI_ISL_2431431	hCoV-19/Brazil/RS-LMM45503/2021
	EPI_ISL_2617875	hCoV-19/Brazil/RS-165_LABRESIS/2020
	EPI_ISL_2617876	hCoV-19/Brazil/RS-166_LABRESIS/2020
	EPI_ISL_2617879	hCoV-19/Brazil/RS-170_LABRESIS/2020
	EPI_ISL_729801	hCoV-19/Brazil/RS-FIOCRUZ-15270/2020
	EPI_ISL_729803	hCoV-19/Brazil/RS-FIOCRUZ-15286/2020
	EPI_ISL_729845	hCoV-19/Brazil/RS-FIOCRUZ-15279/2020
	EPI_ISL_729852	hCoV-19/Brazil/RS-FIOCRUZ-15281/2020
	EPI_ISL_729853	hCoV-19/Brazil/RS-FIOCRUZ-15284/2020
	EPI_ISL_729854	hCoV-19/Brazil/RS-FIOCRUZ-15289/2020

	EPI_ISL_729856	hCoV-19/Brazil/RS-FIOCRUZ-15275/2020	
	EPI_ISL_729861	hCoV-19/Brazil/RS-FIOCRUZ-15292/2020	
	EPI_ISL_770555	hCoV-19/Brazil/RS-00603/2020	
	EPI_ISL_770558	hCoV-19/Brazil/RS-00606/2020	
	EPI_ISL_770562	hCoV-19/Brazil/RS-00610/2020	
	EPI_ISL_770569	hCoV-19/Brazil/RS-00617/2020	
	EPI_ISL_770572	hCoV-19/Brazil/RS-00620/2020	
	EPI_ISL_770573	hCoV-19/Brazil/RS-00622/2020	
	EPI_ISL_770576	hCoV-19/Brazil/RS-00626/2020	
	EPI_ISL_770577	hCoV-19/Brazil/RS-00627/2020	
	EPI_ISL_770582	hCoV-19/Brazil/RS-00634/2020	
	EPI_ISL_770585	hCoV-19/Brazil/RS-00637/2020	
	EPI_ISL_770586	hCoV-19/Brazil/RS-00638/2020	
	EPI_ISL_770588	hCoV-19/Brazil/RS-00640/2020	
	EPI_ISL_770590	hCoV-19/Brazil/RS-00643/2020	
	EPI_ISL_770597	hCoV-19/Brazil/RS-00652/2020	
	EPI_ISL_770599	hCoV-19/Brazil/RS-00654/2020	
	EPI_ISL_770600	hCoV-19/Brazil/RS-00656/2020	
	EPI_ISL_770601	hCoV-19/Brazil/RS-00657/2020	
	EPI_ISL_770608	hCoV-19/Brazil/RS-00666/2020	
	EPI_ISL_770609	hCoV-19/Brazil/RS-00667/2020	
	EPI_ISL_770610	hCoV-19/Brazil/RS-00668/2020	
	EPI_ISL_770611	hCoV-19/Brazil/RS-00669/2020	
	EPI_ISL_770614	hCoV-19/Brazil/RS-00673/2020	
	EPI_ISL_770615	hCoV-19/Brazil/RS-00674/2020	
	EPI_ISL_770623	hCoV-19/Brazil/RS-00685/2020	
	EPI_ISL_770626	hCoV-19/Brazil/RS-00690/2020	
	EPI_ISL_770627	hCoV-19/Brazil/RS-00691/2020	
	EPI_ISL_770629	hCoV-19/Brazil/RS-00693/2020	
	EPI_ISL_779156	hCoV-19/Brazil/RS-00602/2020	
	EPI_ISL_779160	hCoV-19/Brazil/RS-00641/2020	
	EPI_ISL_779161	hCoV-19/Brazil/RS-00644/2020	
	EPI_ISL_779162	hCoV-19/Brazil/RS-00658/2020	
	EPI_ISL_779163	hCoV-19/Brazil/RS-00664/2020	
	EPI_ISL_779165	hCoV-19/Brazil/RS-00677/2020	

	EPI_ISL_779166	hCoV-19/Brazil/RS-00678/2020
	EPI_ISL_779167	hCoV-19/Brazil/RS-00687/2020
	EPI_ISL_779168	hCoV-19/Brazil/RS-00688/2020
	EPI_ISL_831688	hCoV-19/Brazil/RS-15371/2020
	EPI_ISL_831689	hCoV-19/Brazil/RS-19337/2020
	EPI_ISL_831938	hCoV-19/Brazil/RS-24565/2020
	EPI_ISL_832009	hCoV-19/Brazil/RS-27623/2020
	EPI_ISL_832011	hCoV-19/Brazil/RS-31786/2020
	EPI_ISL_930857	hCoV-19/Brazil/RS-33704/2020
	EPI_ISL_942898	hCoV-19/Brazil/RS-38343/2020
	EPI_ISL_943581	hCoV-19/Brazil/RS-36765/2020
	EPI_ISL_943584	hCoV-19/Brazil/RS-36850/2020
	EPI_ISL_943606	hCoV-19/Brazil/RS-82912/2020
	EPI_ISL_943609	hCoV-19/Brazil/RS-83041/2020
Dataset C	EPI_ISL_1034304	hCoV-19/Brazil/AM-FIOCRUZ-21140415MR-R2/2021
	EPI_ISL_1034306	hCoV-19/Brazil/AM-FIOCRUZ-21140646MJ-R2/2021
	EPI_ISL_1068110	hCoV-19/Brazil/AM-FIOCRUZ-20143088ER/2020
	EPI_ISL_1068111	hCoV-19/Brazil/AM-FIOCRUZ-20143093FL/2020
	EPI_ISL_1068112	hCoV-19/Brazil/AM-FIOCRUZ-20143105JJ/2020
	EPI_ISL_1068114	hCoV-19/Brazil/AM-FIOCRUZ-20143297CR/2020
	EPI_ISL_1068149	hCoV-19/Brazil/AM-FIOCRUZ-20843185MR/2020
	EPI_ISL_1068150	hCoV-19/Brazil/AM-FIOCRUZ-20843187NC/2020
	EPI_ISL_1068151	hCoV-19/Brazil/AM-FIOCRUZ-20843198JS/2020
	EPI_ISL_1068154	hCoV-19/Brazil/AM-FIOCRUZ-20843278FR/2020
	EPI_ISL_1068156	hCoV-19/Brazil/AM-FIOCRUZ-20843312KD/2020
	EPI_ISL_1068157	hCoV-19/Brazil/AM-FIOCRUZ-20843348JS/2020
	EPI_ISL_1068158	hCoV-19/Brazil/AM-FIOCRUZ-20843351MC/2020
	EPI_ISL_1068159	hCoV-19/Brazil/AM-FIOCRUZ-20843355YM/2020
	EPI_ISL_1068160	hCoV-19/Brazil/AM-FIOCRUZ-20843374ML/2020
	EPI_ISL_1068169	hCoV-19/Brazil/AM-FIOCRUZ-20890057CR/2020
	EPI_ISL_1068198	hCoV-19/Brazil/AM-FIOCRUZ-20891068DS/2020
	EPI_ISL_1068221	hCoV-19/Brazil/AM-FIOCRUZ-20892199FT/2020
	EPI_ISL_1068222	hCoV-19/Brazil/AM-FIOCRUZ-20892204FL/2020
	EPI_ISL_1068225	hCoV-19/Brazil/AM-FIOCRUZ-20892251AR/2020
	EPI_ISL_1068226	hCoV-19/Brazil/AM-FIOCRUZ-20892262DR/2020

	EPI_ISL_1068243	hCoV-19/Brazil/AM-FIOCRUZ-20893866MN/2020
	EPI_ISL_1068248	hCoV-19/Brazil/AM-FIOCRUZ-20894927MS/2020
	EPI_ISL_1068249	hCoV-19/Brazil/AM-FIOCRUZ-20895217CM/2020
	EPI_ISL_1068256	hCoV-19/Brazil/AM-FIOCRUZ-20897269OP/2020
	EPI_ISL_1068258	hCoV-19/Brazil/AM-FIOCRUZ-21142481RG/2021
	EPI_ISL_1068260	hCoV-19/Brazil/AM-FIOCRUZ-21143321SM/2021
	EPI_ISL_1068261	hCoV-19/Brazil/AM-FIOCRUZ-21143333CC/2021
	EPI_ISL_1068262	hCoV-19/Brazil/AM-FIOCRUZ-21143339MM/2021
	EPI_ISL_1068263	hCoV-19/Brazil/AM-FIOCRUZ-21143395RC/2021
	EPI_ISL_1068264	hCoV-19/Brazil/AM-FIOCRUZ-21143402KJ/2021
	EPI_ISL_1068266	hCoV-19/Brazil/AM-FIOCRUZ-21143420WC/2021
	EPI_ISL_1068268	hCoV-19/Brazil/AM-FIOCRUZ-21143455VM/2021
	EPI_ISL_1068269	hCoV-19/Brazil/AM-FIOCRUZ-21143459HF/2021
	EPI_ISL_1068270	hCoV-19/Brazil/AM-FIOCRUZ-21143496CM/2021
	EPI_ISL_1068271	hCoV-19/Brazil/AM-FIOCRUZ-21143504VA/2021
	EPI_ISL_1068272	hCoV-19/Brazil/AM-FIOCRUZ-21143537SL/2021
	EPI_ISL_1068273	hCoV-19/Brazil/AM-FIOCRUZ-21143541HC/2021
	EPI_ISL_1068274	hCoV-19/Brazil/AM-FIOCRUZ-21143575RS/2021
	EPI_ISL_1068275	hCoV-19/Brazil/AM-FIOCRUZ-21143580DO/2021
	EPI_ISL_1068276	hCoV-19/Brazil/AM-FIOCRUZ-21143603CF/2021
	EPI_ISL_1068278	hCoV-19/Brazil/AM-FIOCRUZ-21143608MV/2021
	EPI_ISL_1068279	hCoV-19/Brazil/AM-FIOCRUZ-21843439LC/2021
	EPI_ISL_1068280	hCoV-19/Brazil/AM-FIOCRUZ-21843511WC/2021
	EPI_ISL_1068281	hCoV-19/Brazil/AM-FIOCRUZ-21843518RA/2021
	EPI_ISL_1068282	hCoV-19/Brazil/AM-FIOCRUZ-21843667JC/2021
	EPI_ISL_1068283	hCoV-19/Brazil/AM-FIOCRUZ-21843677AF/2021
	EPI_ISL_1068284	hCoV-19/Brazil/AM-FIOCRUZ-21843807PS/2021
	EPI_ISL_1068285	hCoV-19/Brazil/AM-FIOCRUZ-21890002SP/2021
	EPI_ISL_1068286	hCoV-19/Brazil/AM-FIOCRUZ-21890006JG/2021
	EPI_ISL_1068287	hCoV-19/Brazil/AM-FIOCRUZ-21890017IF/2021
	EPI_ISL_1068288	hCoV-19/Brazil/AM-FIOCRUZ-21890017MD/2021
	EPI_ISL_1068289	hCoV-19/Brazil/AM-FIOCRUZ-21890025RM/2021
	EPI_ISL_1068290	hCoV-19/Brazil/AM-FIOCRUZ-21890031RC/2021
	EPI_ISL_1068291	hCoV-19/Brazil/AM-FIOCRUZ-21890365EC/2021
	EPI_ISL_1068292	hCoV-19/Brazil/AM-FIOCRUZ-21890474MD/2021

	EPI_ISL_1166615	hCoV-19/Brazil/RN-IEC-177512/2021
	EPI_ISL_1213190	hCoV-19/Brazil/AM-00701/2021
	EPI_ISL_1213202	hCoV-19/Brazil/AM-00700/2021
	EPI_ISL_1213204	hCoV-19/Brazil/AM-00702/2021
	EPI_ISL_1213206	hCoV-19/Brazil/AM-00703/2021
	EPI_ISL_1239012	hCoV-19/Brazil/AM-FIOCRUZ-IAM1504/2021
	EPI_ISL_1261122	hCoV-19/Brazil/AM-FIOCRUZ-20897281WS/2020
	EPI_ISL_1261123	hCoV-19/Brazil/AM-FIOCRUZ-21840593CL/2021
	EPI_ISL_1261683	hCoV-19/Brazil/AM-IEC-177881/2021
	EPI_ISL_1261685	hCoV-19/Brazil/AM-IEC-177889/2021
	EPI_ISL_1261690	hCoV-19/Brazil/AM-IEC-178078/2021
	EPI_ISL_1261694	hCoV-19/Brazil/AM-IEC-177911/2021
	EPI_ISL_1402431	hCoV-19/Brazil/AM-FIOCRUZ-3521-1P/2021
	EPI_ISL_1708318	hCoV-19/Brazil/AM-M1-CAMPI231I/2020
	EPI_ISL_2241532	hCoV-19/Brazil/PE-BA1200-260461726/2021
	EPI_ISL_2241605	hCoV-19/Brazil/AM-BA1200-260461655/2021
	EPI_ISL_2298850	hCoV-19/Brazil/AM-IEC1200-178727/2020
	EPI_ISL_2298852	hCoV-19/Brazil/AM-IEC1200-178729/2020
	EPI_ISL_2298854	hCoV-19/Brazil/AM-IEC1200-178730/2021
	EPI_ISL_2298856	hCoV-19/Brazil/AM-IEC1200-178732/2021
	EPI_ISL_2298867	hCoV-19/Brazil/AM-IEC1200-178766/2020
	EPI_ISL_2298871	hCoV-19/Brazil/AM-IEC1200-178768/2020
	EPI_ISL_2466265	hCoV-19/Brazil/AM-FIOCRUZ-34077-1P/2020
	EPI_ISL_2488774	hCoV-19/Brazil/AM-IEC-177910/2021
	EPI_ISL_2488775	hCoV-19/Brazil/AM-IEC-178063/2021
	EPI_ISL_2499748	hCoV-19/Brazil/AM-M2-CAMPI311/2021
	EPI_ISL_811149	hCoV-19/Brazil/AM-FIOCRUZ-20143138FN-R2/2020
	EPI_ISL_833136	hCoV-19/Brazil/AM-FIOCRUZ-20143103JT/2020
	EPI_ISL_833137	hCoV-19/Brazil/AM-FIOCRUZ-20842882CA/2020
	EPI_ISL_833138	hCoV-19/Brazil/AM-FIOCRUZ-20843201ML/2020
	EPI_ISL_833139	hCoV-19/Brazil/AM-FIOCRUZ-20843228ZC/2020
	EPI_ISL_833140	hCoV-19/Brazil/AM-FIOCRUZ-20843269RC/2020
	EPI_ISL_833167	hCoV-19/Brazil/AM-987/2020
	EPI_ISL_833169	hCoV-19/Brazil/AM-989/2020
	EPI_ISL_833171	hCoV-19/Brazil/AM-991/2020

	EPI_ISL_833172	hCoV-19/Brazil/AM-992/2020
	EPI_ISL_833173	hCoV-19/Brazil/AM-993/2020
	EPI_ISL_833174	hCoV-19/Brazil/AM-994/2020
	EPI_ISL_906069	hCoV-19/Brazil/AM-1035/2021
	EPI_ISL_906071	hCoV-19/Brazil/AM-1062/2021
	EPI_ISL_906075	hCoV-19/Brazil/AM-1031/2021
	EPI_ISL_906076	hCoV-19/Brazil/AM-1036/2021
	EPI_ISL_906077	hCoV-19/Brazil/AM-1037/2021
	EPI_ISL_906080	hCoV-19/Brazil/AM-1047/2021
	EPI_ISL_906081	hCoV-19/Brazil/AM-1048/2021
	EPI_ISL_918499	hCoV-19/Brazil/AM-IEC-177290/2020
	EPI_ISL_918500	hCoV-19/Brazil/AM-IEC-177291/2020
	EPI_ISL_918501	hCoV-19/Brazil/AM-IEC-177292/2020
	EPI_ISL_918502	hCoV-19/Brazil/AM-IEC-177293/2020
	EPI_ISL_918503	hCoV-19/Brazil/AM-IEC-177294/2020
	EPI_ISL_918504	hCoV-19/Brazil/AM-IEC-177301/2020
	EPI_ISL_918505	hCoV-19/Brazil/AM-IEC-177310/2020
	EPI_ISL_918506	hCoV-19/Brazil/AM-IEC-177312/2020
	EPI_ISL_918507	hCoV-19/Brazil/AM-IEC-177314/2021
	EPI_ISL_918508	hCoV-19/Brazil/AM-IEC-177320/2021
	EPI_ISL_918509	hCoV-19/Brazil/AM-IEC-177322/2021
	EPI_ISL_918510	hCoV-19/Brazil/AM-IEC-177323/2021
	EPI_ISL_918511	hCoV-19/Brazil/AM-IEC-177328/2020
	EPI_ISL_940614	hCoV-19/Brazil/AM-1059/2021
	EPI_ISL_940615	hCoV-19/Brazil/AM-1060/2021
	EPI_ISL_940616	hCoV-19/Brazil/AM-1061/2021
	EPI_ISL_940617	hCoV-19/Brazil/AM-1063/2021
	EPI_ISL_940618	hCoV-19/Brazil/AM-1064/2021
	EPI_ISL_940620	hCoV-19/Brazil/AM-1066/2021
	EPI_ISL_940623	hCoV-19/Brazil/AM-1069/2021
	EPI_ISL_940624	hCoV-19/Brazil/AM-1070/2021
	EPI_ISL_940626	hCoV-19/Brazil/AM-1073/2021
	EPI_ISL_940627	hCoV-19/Brazil/AM-1074/2021
	EPI_ISL_943990	hCoV-19/Brazil/AM-1192/2021
	EPI_ISL_1121316	hCoV-19/Brazil/RS-1769/2021

	EPI_ISL_1630158	hCoV-19/Brazil/RS-LMM38991/2020
	EPI_ISL_2038957	hCoV-19/Brazil/RS-FIOCRUZ-7363/2021
	EPI_ISL_2249369	hCoV-19/Brazil/RS-FUNED-6924-21/2021
Dataset D	EPI_ISL_1821208	hCoV-19/Brazil/SP-2389/2020
	EPI_ISL_2241496	hCoV-19/Brazil/PB-BA1200-250009713/2020
	EPI_ISL_2249440	hCoV-19/Brazil/RJ-FUNED-330105720/2020
	EPI_ISL_2249444	hCoV-19/Brazil/RJ-FUNED-330107714/2020
	EPI_ISL_2344420	hCoV-19/Brazil/PR-L119-CD7789/2020
	EPI_ISL_2614541	hCoV-19/Brazil/GO-2732R1/2021

Supplementary Figures

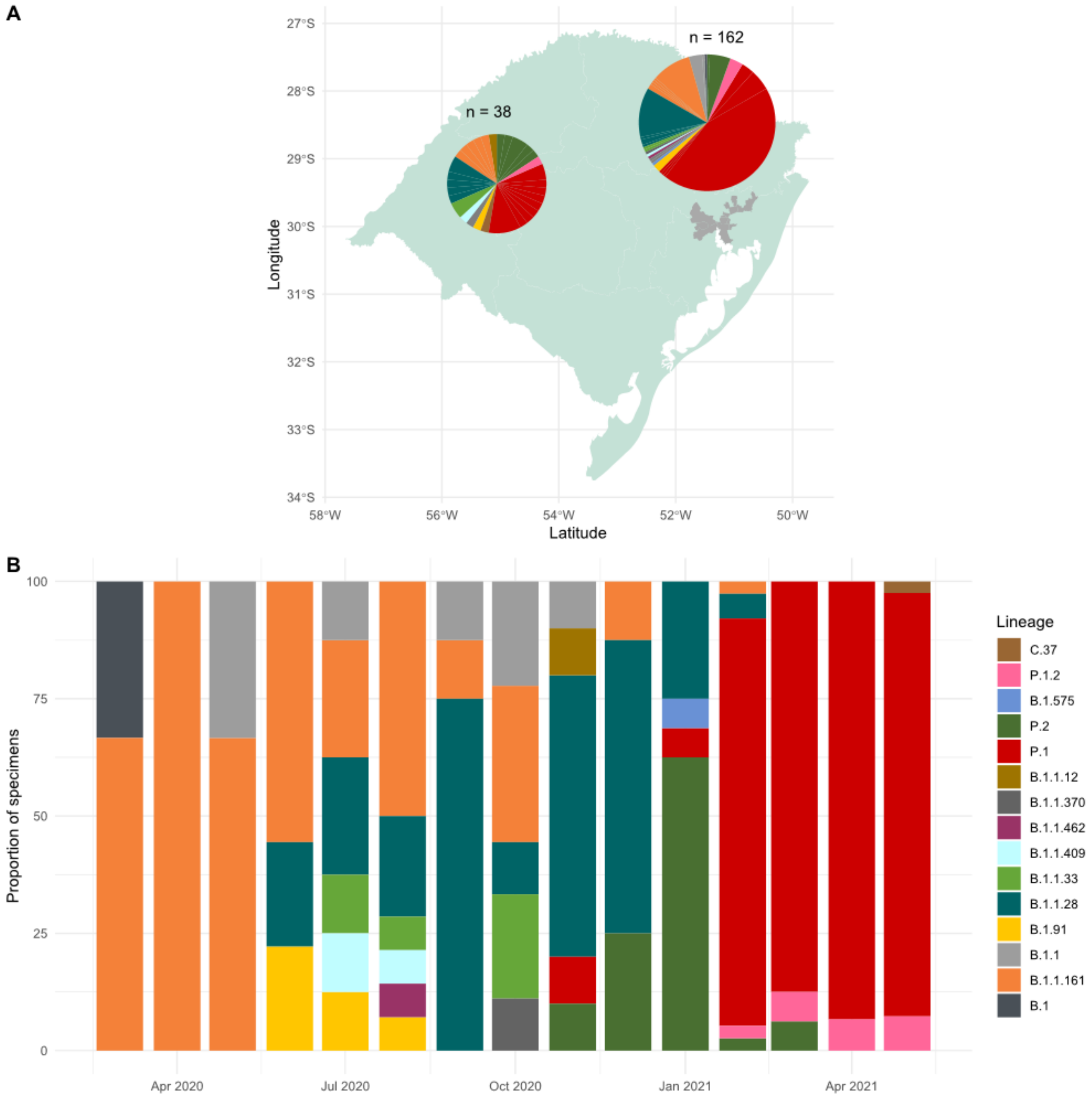

Figure S1. Distribution of SARS-CoV-2 lineages across specimens from COVID-19 cases collected during distinct periods of the study, Rio Grande do Sul state, Southern Brazil, March 2020 to May 2021 (n = 200 specimens). A) Map of Rio Grande do Sul with Porto Alegre and metropolitan area highlighted. B) Distribution of SARS-CoV-2 lineages in Rio Grande do Sul, across the sampling period.

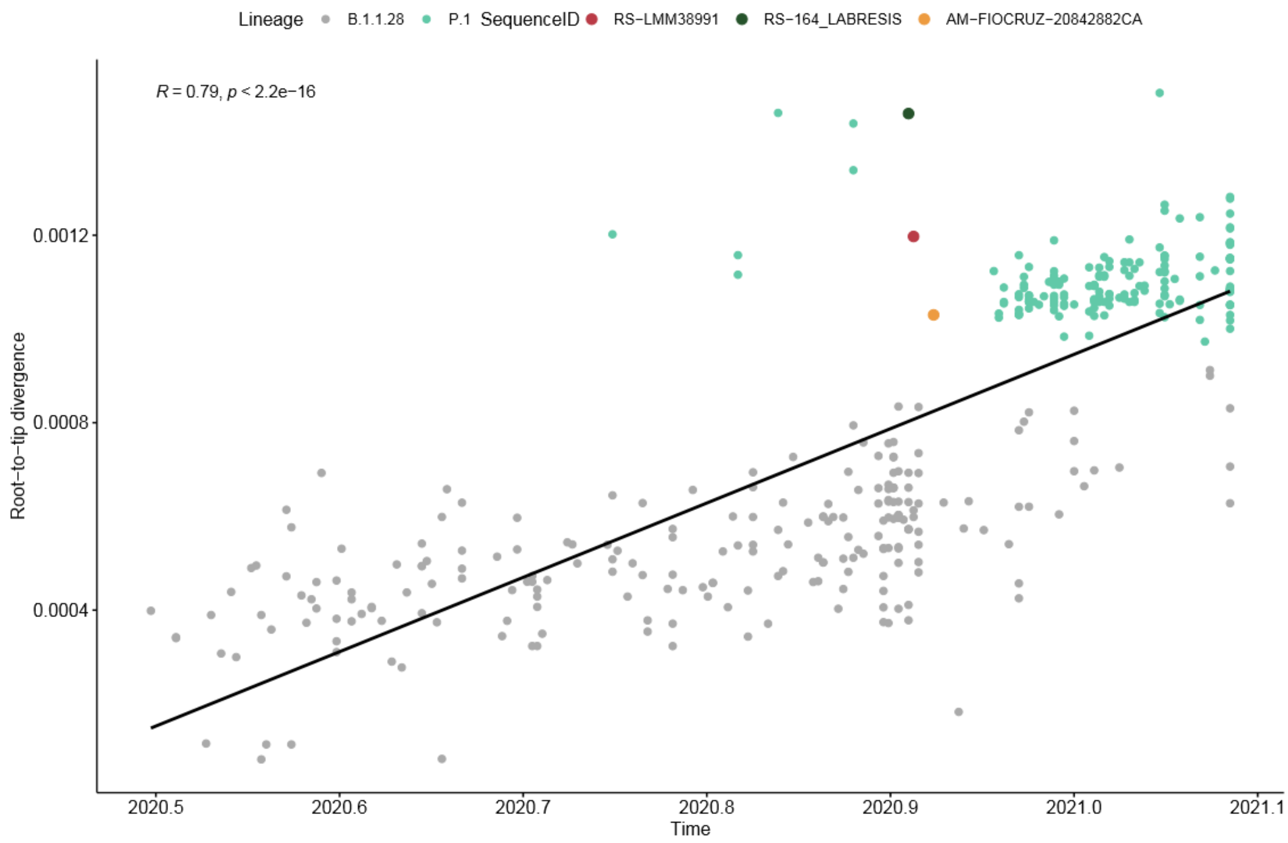

Figure S2. Root-to-tip regression of genetic distances and sampling dates for dataset A, B, C and D (n=389). Correlation coefficient is depicted above the graph. The sequences RS−LMM38991(red) and RS-164_LABRESIS (dark green), both from Rio Grande do Sul, and the sequence AM−FIOCRUZ−20842882CA (orange) from Amazonas are highlighted in different colors as they are the oldest P.1 specimens in their states.

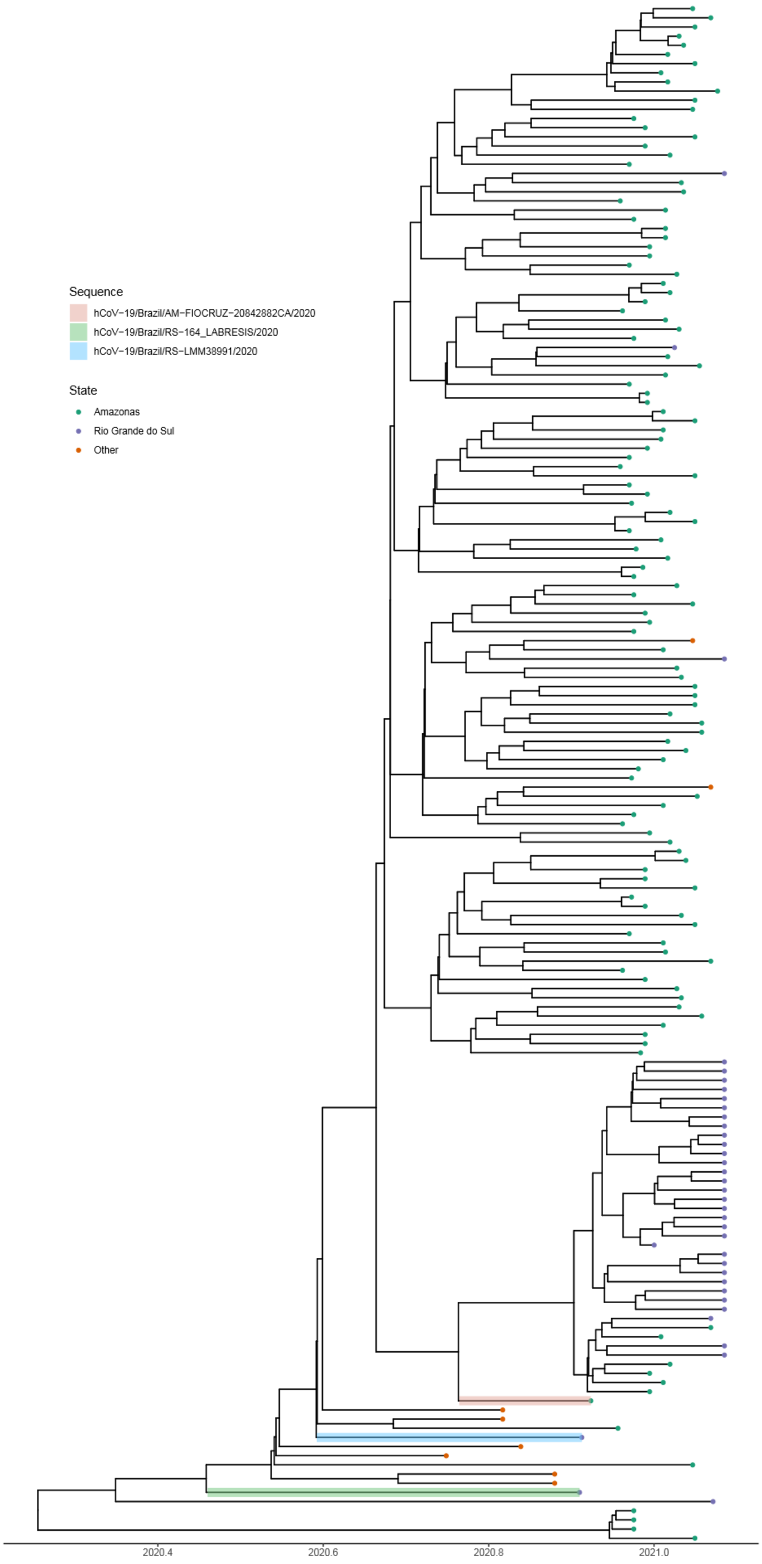

Figure S3. Time-scaled and reconstruction of the maximum clade credibility tree for all datasets in this study (n=389). The B.1.1.28 lineage was collapsed for best visualization. The tip of each sequence was colored to represent the different regions: Amazonas (green), Rio Grande do Sul (purple) and from other states (orange). The sequences 164_LABRESIS (RS) and RS−LMM38991 (EPI_ISL_1630158), both from Southern Brazil, and the sequence AM−FIOCRUZ−20842882CA (EPI_ISL_833137), from Northern Brazil, were highlighted in different colors as they are the older P.1 sequences.

References

1. Wink PL, Volpato F, Lima-Morales D, Paiva RM, Willig JB, Bock H, De-Paris F, Barth AL. 2021. RT-qPCR half reaction optimization for the detection of SARS-CoV-2. medRxiv 05(19)21257470. doi: 10.1101/2021.05.19.21257470
2. Pangolin: lineage assignment in an emerging pandemic as an epidemiological tool. [v3.0.2]. Available from: http://pangolin.cog-uk.io. Accessed 21 July, 2021
3. Rambaut A, Holmes EC, O'Toole A, Hill V, McCrone JT, Ruis C, Plessis L, Pybus OG. 2020. A dynamic nomenclature proposal for SARS-CoV-2 lineages to assist genomic epidemiology. Nat Microbiol 5:1403-1407. doi:10.1038/s41564-020-0770-5
4. Nextclade - Clade assignment, mutation calling, and sequence quality checks [v1.5.2]. Available from: https://clades.nextstrain.org. Accessed 21 July, 2021
5. Minh BQ, Schmidt HA, Chernomor O, Schrempf D, Woodhams MD, Haeseler A, Lanfear R. 2020. IQ-TREE 2: New Models and Efficient Methods for Phylogenetic Inference in the Genomic Era. Mol Biol Evol 37(5):1530-1534. doi: 10.1093/molbev/msaa015
6. Kalyaanamoorthy S, Minh BQ, Wong TKF, von Haeseler A, Jermiin LS. 2017. ModelFinder: fast model selection for accurate phylogenetic estimates. Nat Methods 14:587-589. doi:10.1038/nmeth.4285
7. Rambaut A, Lam TT, Carvalho LM, Pybus OG. 2016. Exploring the temporal structure of heterochronous sequences using TempEst (formerly Path-O-Gen). Virus Evol 2(1)1:7. doi: 10.1093/ve/vew007
8. Bouckaert R, Vaughan TG, Barido-Sottani J, Duchêne S, Fourment M, Gavryushkina A, Heled J, Jones F, Kuhnert D, Mario N, Matschiner M, Mendes FK, müller NF, Ogilvie HA, Plessis L, Popinga A, Rambaut A, Rasmussen D, Siveroni I, Suchard MA, Wu CH, Xie D, Zhang C, Stadler T, Drummond AJ. 2019. BEAST 2.5: An Advanced software platform for Bayesian evolutionary analysis. Plos Comput Biol 15(4):e1006650. doi: 10.1371/ journal.pcbi.1006650
9. Drummond AJ, Rambaut A, Shapiro B, Oybus OG. 2005. Bayesian coalescent inference of past population dynamics from molecular sequences. Mol Biol Evol 22(5):1185-1192. doi:10.1093/molbev/msi103
10. Rambaut A, Drummond AJ, Xie D, Baele G, Suchard MA. 2018. Posterior summarization in Bayesian Phylogenetics using Tracer 1.7. Syst Biol 67(5):901-904. Doi: 10.1093/sysbio/syy032
11. Ishikawa SA, Zhukova A, Iwasaki W, Gascuel O. 2019. A Fast Likelihood Method to Reconstruct and Visualize Ancestral Scenarios. Mol Biol Evol 36:2069–2085. https://doi.org/10.1093/MOLBEV/MSZ131
12. Letunic I, Bork P. Interactive Tree Of Life (iTOL) v4: recent updates and new developments. 2019. Nucleic Acids Res 47:W256–W259. https://doi.org/10.1093/NAR/GKZ239
13. Guangchuang Yu, David Smith, Huachen Zhu, Yi Guan, Tommy Tsan-Yuk Lam. 2017. ggtree: an R package for visualization and annotation of phylogenetic trees with their covariates and other associated data. Methods Ecol and Evol 8(1):28-36. doi:10.1111/2041-210X.12628
14. Wang LG, Lam TTY, Xu S, Dai Z, Zhou L, Feng T, Guo P, Dunn CW, Jones BR, Bradley T, Zhu H, Guan Y, Jiang Y, Yu G. 2020. treeio: an R package for phylogenetic tree input and output with richly annotated and associated data. Mol Biol and Evol 37(2):599-603. doi: 10.1093/molbev/msz240

15. Wickham H. 2016. ggplot2: Elegant Graphics for Data Analysis. Springer-Verlag New York. ISBN 978-3-319-24277-4. Available from: https://ggplot2.tidyverse.org. Accessed 21July, 2021
16. Pereira RHM, Gonçalves CN, et all. 2019. geobr: Loads Shapefiles of Official Spatial Data Sets of Brazil. Available from: GitHub repository - https://github.com/ipeaGIT/geobr. Accessed 21 July, 2021
17. Wickham H, François R, Henry L, Müller K. 2021. dplyr: A Grammar of Data Manipulation. R package version 1.0.6. Available from: https://CRAN.R-project.org/package=dplyr. Accessed 21 July, 2021
